# Metabolomics integrated with machine learning to discriminate the geographic origin of Rougui Wuyi rock tea

**DOI:** 10.1038/s41538-023-00187-1

**Published:** 2023-03-16

**Authors:** Yifei Peng, Chao Zheng, Shuang Guo, Fuquan Gao, Xiaxia Wang, Zhenghua Du, Feng Gao, Feng Su, Wenjing Zhang, Xueling Yu, Guoying Liu, Baoshun Liu, Chengjian Wu, Yun Sun, Zhenbiao Yang, Zhilong Hao, Xiaomin Yu

**Affiliations:** 1 College of Horticulture, Fujian Agriculture and Forestry University, Fuzhou, 350002 China; 2 FAFU-UCR Joint Center for Horticultural Biology and Metabolomics, Haixia Institute of Science and Technology, Fujian Agriculture and Forestry University, Fuzhou, 350002 China; 3 Fujian Farming Technology Extension Center, Fuzhou, 350003 China; 4 Wuyishan Institute of Agricultural Sciences, Wuyishan, 354300 China; 5 Wuyishan Tea Bureau, Wuyishan, 354300 China; 6 Fujian Vocational College of Agriculture, Fuzhou, 350119 China

**Keywords:** Metabolomics, Metabolomics, Metabolomics

## Abstract

The geographic origin of agri-food products contributes greatly to their quality and market value. Here, we developed a robust method combining metabolomics and machine learning (ML) to authenticate the geographic origin of Wuyi rock tea, a premium oolong tea. The volatiles of 333 tea samples (174 from the core region and 159 from the non-core region) were profiled using gas chromatography time-of-flight mass spectrometry and a series of ML algorithms were tested. Wuyi rock tea from the two regions featured distinct aroma profiles. Multilayer Perceptron achieved the best performance with an average accuracy of 92.7% on the training data using 176 volatile features. The model was benchmarked with two independent test sets, showing over 90% accuracy. Gradient Boosting algorithm yielded the best accuracy (89.6%) when using only 30 volatile features. The proposed methodology holds great promise for its broader applications in identifying the geographic origins of other valuable agri-food products.

## Introduction

With the rapid growth in international trade and raising awareness among consumers of the importance of food safety, issues around food authenticity have been receiving increased public attention in recent years^1^. Consumers have become increasingly concerned about what foods to buy, where they are from and how they are produced. Agricultural and food products derived from certain core production regions are perceived to have a higher quality and hence a higher market value. Despite strict legislative control, adulteration and false labeling are still prevalent in the food industry driven by economic incentives. This further aggravates unfair market competition, reduces consumer trust, and leads to potential risks to the public health^2,3^. Therefore, the effective identification of the geographic origin of foodstuffs is essential for ensuring food quality and protecting consumer interest.

Tea (*Camellia sinensis*) is one of the most widely consumed beverages in the world, due to an array of nutritional and health benefits. Regional provenance is among the most important attributes that tea consumers associate with high-quality tea products^4–8^. Many teas are named based on the geographic regions where they are grown. The most prominent examples are Darjeeling from India, Ceylon from Sri Lanka as well as Westlake Longjing tea and Wuyi rock tea from China. These tea products are highly sought after by tea enthusiasts, the fact that also makes them prime targets for fraud.

For tea authentication, a variety of analytical tools have been developed, such as stable isotope analysis, multi-element profiling and metabolite fingerprinting^9^. The first two methods, used alone or more often in combination, are acknowledged as being effective for the authentication of a wide range of food products including tea^4,10,11^. However, both measurements require laborious sample preparation and are hence technically demanding. Metabolomics in conjunction with chemometrics has emerged as one of the most promising methods to distinguish tea origins because of a wide metabolite coverage, high sensitivity and high throughput^5–8,12,13^. In particular, gas chromatography-mass spectrometry (GC-MS), a technique well-recognized for its capability to simultaneously monitor a large number of volatile organic compounds (VOCs) with proven reproducibility, has become a central platform for tea metabolomics research^13–18^. Solid phase microextraction (SPME), which integrates sampling, volatile extraction and concentration in one single step, greatly simplifies the sample preparation and thus is widely adopted in food analysis^19^. In recent years, SPME in coupled with GC-MS has been successfully applied to assess the geographic origins of various food products, such as rice^20,21^, wine^22^ and oranges^23^. As such, VOC fingerprinting by SPME-GC-MS represents a promising approach to identify the geographic origin of tea.

In the past decade, machine learning (ML), which is capable of generating high accuracy outputs from massive volumes of multidimensional data, has found wide applications in many domains of science for pattern recognition, classification and prediction^24–26^. In particular, its surging popularity for food authentication, origin identification and quality control is amply demonstrated by the increasing number of publications on this topic over the past few years, highlighting the great potential for food inspection and classification^27–32^. Although metabolomics has been widely applied in tea research, the application of ML techniques in their analyses has just emerged^5,13^. Nonetheless, the sample sizes in existing studies are typically small. As the performance of a specific classifier could be greatly influenced by the sample size, a small sample set is prone to errors in pattern recognition. Moreover, not enough efforts have been dedicated to distinguishing tea from a narrow geographic scope.

Wuyi rock tea (WRT), belonging to a preeminent subcategory of oolong tea, is internationally renowned for intriguing flavors and lingering taste with a heavy roast note^33,34^. WRT has been granted protected geographical indication (PGI) status in China, which in the broad term includes all oolong tea produced in Wuyishan City of Fujian Province using suitable tea varieties and manufactured by the unique traditional processing technology. The distinctive flavor of WRT is decided by tea cultivars, the growth environment and the manufacturing procedures^35^. More than 200 tea cultivars are used to manufacture WRT, in which “Rougui” and “Shuixian” occupy the largest planting area. In practice, WRT is further classified based on the growing location. The core production region features a small area of only about 60 kilometers inside of the national preserve of Mount Wuyi, which is a UNESCO World Heritage Site located in the Northwest Fujian^4^. WRT produced in this region (also known as “Zhengyan”) is perceived to have higher quality, referred locally as “rock charm and floral fragrance” or “rock rhythm”^36,37^. What makes “Zhengyan” WRT so special is the unique terroir where it grows-volcano rock, steep cliff, mineral rich soil, abundant rainfall and high humidity-creating a perfect natural environment for tea cultivation that no other place could find. As expected, WRT produced in the core region usually fetches a high price tag. Due to the conflict between high demand and limited production in the core area, there are many imitations on the market, most of which are grown in areas surrounding the authentic core region and are considered to be inferior in terms of tea quality^4^. The conventional method for quality evaluation of WRT, like other tea, relies heavily on the experience of tea tasters, which not only suffers from inconsistency and inaccuracy, but also lacks quantitative information^38^. For this reason, the development of rapid and reliable analytical methods to trace the geographic origin of WRT is highly demanded.

In this study, we explored the feasibility of ML-based analyses of VOC metabolomes to differentiate the origins of Rougui WRT. Our results show that our ML-based methods accurately authenticate the geographic origin of WRT based on volatile metabolites. The proposed methodology will be useful for managing adulteration or fraudulent labeling of WRT in the market and may be broadly applicable to tracing the origins of other valuable food and agricultural products.

## Results and discussion

### Overview of VOCs of WRT from different origins

**Fig. 1:**
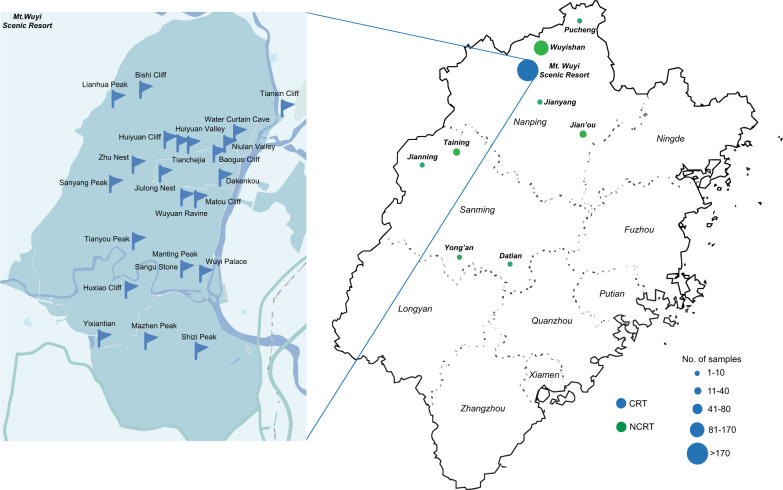
Geographical distributions of sampling points in the current study. The enlarged part indicates representative sampling points located in Mount Wuyi Scenic Resort.

SPME was combined with gas chromatography-time-of-flight mass spectrometry (GC-TOFMS) to identify and quantify VOCs of 333 Rougui WRT samples collected from the main growing regions in the north and northwestern parts of Fujian Province, China (Fig. 1; Supplementary Table S1). Representative total ion chromatograms (TICs) of WRT samples from the core production region (hitherto referred as “CRT”) and the non-core production region (hitherto referred as “NCRT”) are depicted in Supplementary Fig. S1. We detected a total of 2128 features by GC-MS analysis. QC samples, prepared by pooling equal aliquots of all samples, were used to monitor the system performance. Principle component analysis (PCA) performed on all samples showed that QC samples were in a tight cluster in the center of the plot, indicating that the established GC-MS method was stable enough to allow for the subsequent analysis (Supplementary Fig. S2). After applying hierarchical cluster analysis to remove outliers, 276 samples were retained. A final data matrix composed of 447 metabolic features was obtained after eliminating those with low variance (mean absolute deviation ≤0) and was subsequently used for multivariate analysis. Among these, 44 volatiles were identified in comparison with authentic standards and 236 volatiles were tentatively identified by comparing their mass spectra and retention indices with those recorded in the database (NIST 20) or literatures (Supplementary Table S2).

**Fig. 2:**
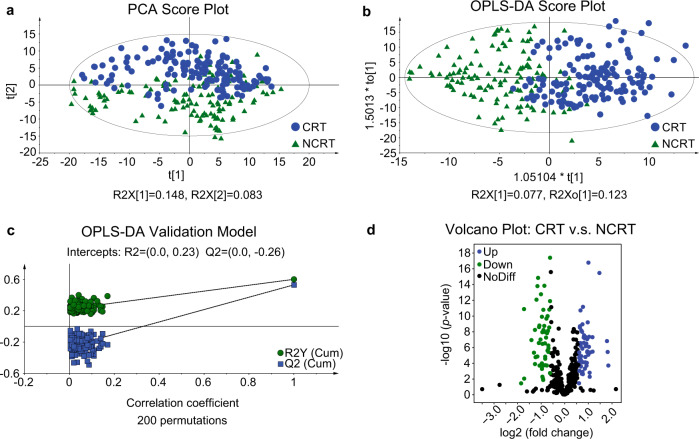
Multivariate statistical analysis of volatile metabolites of CRT and NCRT Wuyi rock tea samples. **a** PCA score plot. **b** OPLS-DA score plot. *R*^*2*^Y_cum_ = 0.602, *Q*^*2*^_cum_ = 0.532. **c** Cross-validation plot of the OPLS-DA model with 200 permutation tests. **d** Volcano plot showing differential metabolites. Blue dots represent fold-change >1.5 and *p* < 0.05. Green dots represent fold-change <0.67 and *p* < 0.05. Black dots represent no statistically significant difference.

No clear clustering of tea samples according to their geographic regions was evident in the PCA score plot, with PC1 and PC2 combined affording only 23.1% of the total variance (Fig. 2a). Orthogonal partial least squares-discriminant analysis (OPLS-DA) has shown to generally outperform the unsupervised PCA method in classifying various agri-products^39–43^. Therefore, it was applied in the current study. As expected, a better separation between groups was achieved by this method (Fig. 2b). The permutation test generated intercepts of *R*^2^ = 0.23 and *Q*^2^ = −0.26, thus confirming the validity of the OPLS-DA model (Fig. 2c).

### Differential VOCs between CRT and NCRT samples

**Fig. 3:**
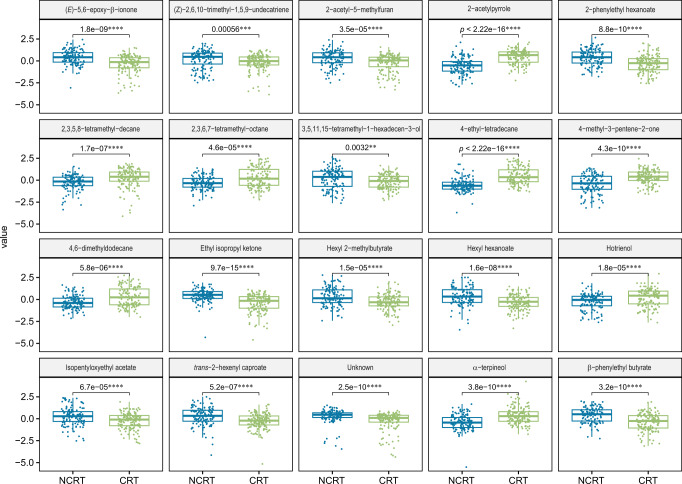
Boxplots showing the levels of differential volatile metabolites between CRT and NCRT Wuyi rock tea samples based on Wilcoxon test. The relative abundance of volatiles with significant differences was presented using log10-transformed Z-score values. **p < = 0.01, ***p < =0.001, ****p < = 0.0001. The box represents the interquartile range, with the median shown as the horizontal line inside the box. The bottom and top boundaries of the box represent the 25th and 75th percentiles, while the lower and upper whiskers correspond to the 5th and 95th percentiles.

The volcano plot analysis highlighted 111 differential VOCs (57 up and 54 down) for the CRT collection in comparison with NCRT (Fig. 2d). Ultimately, 20 VOCs with significant differences, including esters (6), hydrocarbons (5), ketones (3), alcohols (3), heterocycles (2) and one unknown odorant, were identified based on the criteria of variable importance in projection (VIP) > 1, *p* < 0.05 and |fold change | >1.5 (Table 1; Fig. 3).

**Table 1:** Differential volatile compounds tentatively identified in CRT and NCRT Wuyi rock tea samples.

No.	VIP	Compound	CAS No.	Formula	Metabolite class	RT (min)	Odor description	RI_exp_	RI_lit_	Identification method
1	1.78	Ethyl isopropyl ketone	565-69-5	C_6_H_12_O	ketones	3.4	Minty	NA	745	MS, Std
2	1.64	4-methyl-3-penten-2-one	141-79-7	C_6_H_10_O	ketones	4.3	Honey-like, floral	NA	798	MS, Std
3	1.04	2-acetyl-5-methylfuran	1193-79-9	C_7_H_8_O_2_	heterocycles	14.2	Nutty, caramellic	1028	1039	MS, RI, Std
4	1.95	2-acetylpyrrole	1072-83-9	C_6_H_7_NO	heterocycles	16.0	Nutty, bread	1064	1063	MS, RI, Std
5	1.24	Hotrienol	29957-43-5	C_10_H_16_O	alcohols	17.9	Floral, fruity	1102	1107	MS, RI
6	1.17	Unknown	NA	NA	NA	19.0	NA	1125	NA	MS
7	1.61	α-terpineol	98-55-5	C_10_H_18_O	alcohols	22.3	Pine, citrus, woody	1191	1189	MS, RI, Std
8	1.14	Isopentyloxyethyl acetate	204652-53-9	C_9_H_18_O_3_	esters	23.4	NA	1215	1176	MS, RI
9	1.27	Hexyl 2-methylbutyrate	10032-15-2	C_11_H_22_O_2_	esters	24.3	Sweet, fruity	1234	1236	MS, RI, Std
10	1.45	2,3,6,7-tetramethyloctane	52670-34-5	C_12_H_26_	hydrocarbons	26.1	Alkane-like	1273	NA	MS
11	1.55	4,6-dimethyldodecane	61141-72-8	C_14_H_30_	hydrocarbons	28.2	Alkane-like	1319	1325	MS, RI
12	1.13	2,3,5,8-tetramethyldecane	192823-15-7	C_14_H_30_	hydrocarbons	29.0	Alkane-like	1337	1318	MS, RI
13	1.46	Hexyl hexanoate	6378-65-0	C_12_H_24_O_2_	esters	31.0	Fruity, green	1383	1384	MS, RI, Std
14	1.30	trans-2-hexenyl caproate	53398-86-0	C_12_H_22_O_2_	esters	31.1	Fruity, green	1384	1391	MS, RI, Std
15	1.77	β-phenylethyl butyrate	103-52-6	C_12_H_16_O_2_	esters	33.1	Fruity, floral, green	1432	1444	MS, RI, Std
16	1.52	(*E*)-5,6-epoxy-β-ionone	23267-57-4	C_13_H_20_O_2_	ketones	33.2	Fruity, woody, floral	1434	1455	MS, RI
17	1.08	(*Z*)-2,6,10-trimethyl-1,5,9-undecatriene	62951-96-6	C_14_H_24_	hydrocarbons	34.1	NA	1454	NA	MS
18	2.13	4-ethyl-tetradecane	55045-14-2	C_16_H_34_	hydrocarbons	37.5	Alkane-like	1541	1548	MS, RI
19	1.78	2-phenylethyl hexanoate	6290-37-5	C_14_H_20_O_2_	esters	41.1	Fruity, green	1633	1650	MS, RI, Std
20	1.11	3,5,11,15-tetramethyl-1-hexadecen-3-ol	649699-11-6	C_20_H_40_O	alcohols	49.6	NA	1874	NA	MS

*MS* mass spectrum comparison using NIST libraries, *RI* the retention index compared with the literature value, *Std* mass spectrum compared with the chemical reference standard, *NA* not available.

The amounts of volatiles with floral, fruity and woody scents (i.e., hortrienol, *α*-terpineol and 4-methyl-3-penten-2-one) differed mostly between CRT and NCRT (Fig. 3). Generated by the Maillard reaction^44^, 2-acetylpyrrole with a nutty and bread note was also found at a higher level in CRT. Such nitrogen-containing heterocyclic compounds with pyrrole and pyrazine structures largely account for the characteristic aromas in WRT resulting from roasting^37,45^. Additionally, several odorless branched alkanes (i.e., 2,3,6,7-tetramethyloctane, 4,6-dimethyldodecane, 2,3,5,8-tetramethyldecane and 4-ethyl-tetradecane) accumulated to higher levels in CRT than in NCRT, presumably originating from the thermal degradation of terpenes during tea processing^46^.

In contrast, esters that exhibited significant differences were exclusively present in higher proportion in NCRT (Fig. 3). Of note, most of them (i.e., hexyl 2-methylbutyrate, hexyl hexanoate, *trans*-2-hexenyl caproate and *β*-phenylethyl butyrate) impart fruity and green flavor. Likewise, a higher accumulation of 5,6-epoxy-*β*-ionone (fruity and floral) and ethyl isopropyl ketone (minty) was noted in NCRT. In a most recent study, sixteen VOCs (mostly aldehydes and terpenes) were shown to differ among Rougui WRT collected from different cultivation regions^47^. These volatiles, however, have little overlap with what we discovered in the current study, most likely due to the fact that only three tea samples were analyzed in their study and thus were not representative of WRT in general.

Taken together, we can conclude that CRT and NCRT present clearly distinct aroma profiles, with a stronger floral, woody and roasted notes in the former group while stronger fruity and green odors in the latter group. It should be noted that roasting, which is a critical process unique to the processing of WRT, is essential for transforming tea aroma from being floral to roasted and woody^37^. In this regard, it is possible that the differences in the volatile concentrations of CRT and NCRT are not totally due to differences in geographical locations, but may also be influenced by the tea processing procedures. Furthermore, other factors such as climatic conditions, years of harvest and agricultural practices obviously have a certain influence, as demonstrated in similar studies conducted for other agri-products^29,32,48^. Further studies are needed to determine which factor exerts the largest impact on the individual component of WRT aromas.

### Authentication of the origin of WRT with machine learning

**Fig. 4:**
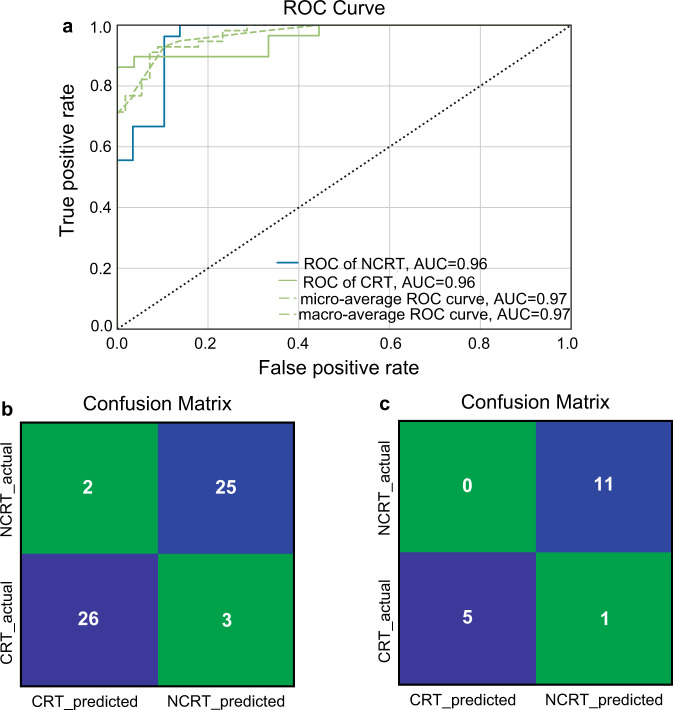
Performance of the multilayer perceptron (MLP) model for provenance discrimination of WRT using 176 volatile features as the input dataset. **a** Receiver operating characteristic (ROC) curve of MLP where the model was trained using 5-fold cross-validation. **b** Confusion matrix of the MLP model on the test set. **c** Confusion matrix of the MLP model on the validation set.

To develop a model for the origin discrimination of WRT, we selected 176 volatile features that could be stably detected across different batches of tea samples for model training (Supplementary Table S2). The dataset was randomly split into two parts in a stratified fashion: 80% (220) for model training and 20% (56) for model validation. Although ML algorithms such as random forest (RF) and support vector machines (SVM) have been widely employed to discriminate the geographic origins of various agricultural products, no single ML method could perform best on all the datasets, given that the meta-features (i.e., statistics about the number of data points, features, classes as well as data skewness and the entropy of the targets) vary across datasets. In addition, the complexity of different ML algorithms varies. To generate the optimal model with the highest prediction accuracy and generalization ability, we tested 15 classification algorithms of Scikit-learn on the VOC metabolomics data to match model and data complexities in our study (Supplementary Dataset 1). Based on five-fold cross-validation, multilayer perceptron (MLP) achieved the best performance, yielding a prediction accuracy of 92.7% (Table 2). MLP, as the name suggests, consists of interconnected neurons that process data through three or more layers. Belonging to a class of feedforward artificial neural networks (ANN), MLP is well recognized for the prediction power and is widely used to extract information from complex and nonlinear relationships^49^. From the ROC (receiver operating characteristic) curve, the AUC (area under the curve) value was computed as 0.96, which validated the generalization ability of the MLP model (Fig. 4a). High classification accuracies (>85%) could also be achieved by other classifiers, especially quadratic discriminant analysis (QDA), passive aggressive (PA) and SVM (Table 2).

**Table 2:** Comparison of the prediction results with different models.

Classifier	Prediction accuracy	
	176 features	30 features
Multilayer Perceptron (MLP)	92.7%	83.2%
Quadratic Discriminant Analysis (QDA)	89.6%	84.6%
Passive Aggressive (PA)	89.6%	83.2%
Support Vector Machines (SVM)	88.6%	83.2%
Linear Discriminant Analysis (LDA)	87.7%	85.5%
Random Forest (RF)	87.7%	86.8%
Stochastic Gradient Descent (SGD)	87.7%	84.6%
Gradient Boosting (GB)	86.8%	89.6%
Adaboost (AB)	86.4%	85.9%
K-Nearest Neighbors (KNN)	86.4%	86.8%
Linear Support Vector Machines (LinearSVM)	85.9%	83.6%
Bernoulli Naive Bayes (BernoulliNB)	85.0%	82.7%
Extra Tree (ET)	84.1%	86.4%
Gaussian Naive Bayes (GaussianNB)	83.2%	84.1%
Decision Tree (DT)	79.1%	82.7%

Given the promising performance of MLP on internal cross validation, a test set was built with the remaining 20% of samples (56 in total) for external validation. About 91.1% of tea samples from the test set were correctly classified (Fig. 4b). To further benchmark the model performance, we then performed an independent validation on a separate set of 17 Rougui WRT samples purchased from the market, leading to 94.1% prediction accuracy (Fig. 4c). Overall, these results unequivocally demonstrate the effectiveness of VOC metabolomics in tandem with ML-based algorithms for provenance discrimination of WRT. By applying SVM modeling to the combined data from isotope ratio mass spectrometry (IRMS) and inductively coupled plasma mass spectrometry (ICP-MS), Lou and coworkers achieved 97.7% accuracy when discriminating between WRT and non-WRT^4^. Since IRMS and ICP-MS are both time-consuming and costly, the proposed metabolomics approach in the current study offers advantages in terms of simplicity and speed while still maintaining comparable accuracy. It serves as a good alternative for the authentication of WRT.

### Machine learning-based prediction model with a simplified input dataset

**Fig. 5:**
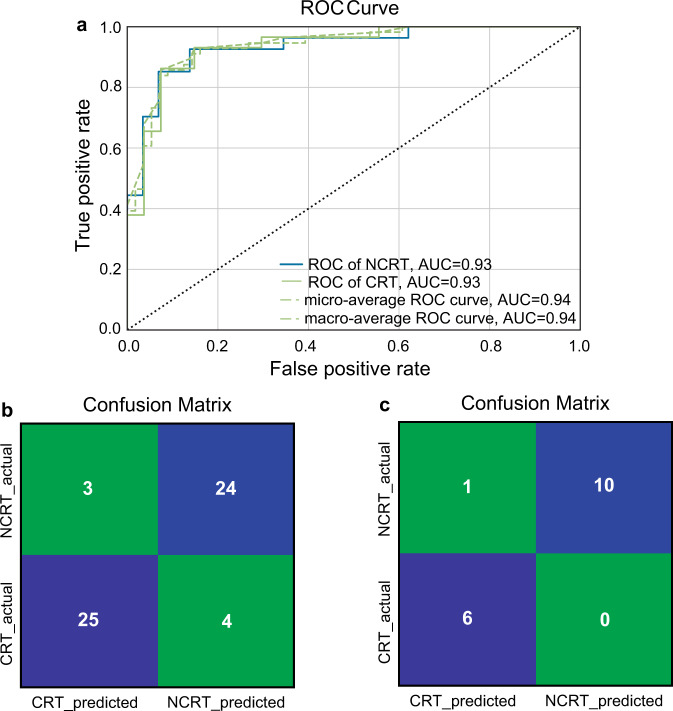
Performance of the gradient boosting (GB) model for provenance discrimination of WRT using the simplified input dataset. **a** ROC curve of GB where the model was trained using 5-fold cross-validation. **b** Confusion matrix of the GB model on the test set. **c** Confusion matrix of the GB model on the validation set.

In an effort to reduce computing power while improving prediction efficiency, a further attempt was made to establish a ML prediction model using a simplified input dataset. Towards this goal, the top 30 metabolic features with the highest VIP scores in the OPLS-DA analysis, instead of the original 176 metabolic features, were included to construct a new model. As the meta-features of the simplified dataset were changed, we tested the aforementioned 15 ML algorithms again to match the model and data complexities (Supplementary Dataset 2). For the simplified dataset, the GB model demonstrated the highest accuracy (89.6%) and AUC values (0.93) among all tested algorithms, slightly higher than 86.8% accuracy obtained by the same model on the original dataset (Table 2; Fig. 5a). Furthermore, 87.5% and 94.1% accuracies were attained on the test and validation sets, respectively, indicating that a satisfactory prediction performance could still be realized with a much-reduced dataset (Fig. 5b, c). Although the MLP model produced an acceptable accuracy (83.2%) for the training set, accuracies for the test sets dropped to below 80%, implying that overfitting may occur and thus degrade the prediction performance. This is not unexpected, given that MLP is prone to overfitting when adopted for the unknown samples and hence usually requires a large number of parameters and extended data training in order to achieve a good generalization on the test set^50^. In contrast, the GB algorithm is typically optimal for small datasets and has proven to outperform deep learning when the training data is not sufficient^51^. Therefore, GB is more advantageous than MLP regarding the overfitting risk and robustness when a simplified dataset is considered.

In summary, the present study demonstrates the geographic origin discrimination of 333 WRT samples, the largest panel tested as far as we know, using a strategy combining VOC fingerprinting with ML. The predictive modeling of VOC metabolomics data has proven to be a highly promising and powerful tool to confirm the authenticity, quality, and origin of WRT. Furthermore, we clearly demonstrate a terroir impact on the flavor of WRT and 20 VOCs are identified to distinguish CRT from NCRT. It should be noted that VOC profiling could be affected by several factors, such as tea storage conditions and processing methods. Moreover, fraud cases in real-life scenarios are even more complicated. For example, intentionally mixing or replacing part of CRT with NCRT for increased commercial value likely happens. Although ML is routinely used to analyze individual chemical datasets, integration of additional spectra data, such as those acquired by liquid-chromatography mass-spectrometry (LC-MS), nuclear magnetic resonance (NMR) or near-infrared (NIR) spectroscopy, as well as prior knowledge on tea samples (i.e., storage conditions and processing methods) will further increase both the accuracy and the generalization ability of the predictive models.

## Methods

### Sample collection

During the periods of 2019 and 2020, a total of 333 authentic WRT (*Camellia sinensis* (L.) O. Kuntze cv. “Rougui”) samples of different origins were collected from various producers in Nanping, the north production area and Sanming, the north-western production area of Fujian Province (Supplementary Table S1). These are labeled as CRT and NCRT, respectively. Sample numbers were as follows: CRT (*n* = 174) and NCRT (*n* = 159). Samples were stored in airtight and lightproof aluminum foil packages at 4 °C prior to analysis.

### Standards

2-acetylpyrrole and *n*-alkane standard solution C_8_-C_25_ were purchased from Sigma-Aldrich (St. Louis, MO, USA). 2-acetyl-5-methylfuran, *β*-phenylethyl butyrate, 2-phenylethyl hexanoate, ethyl isopropyl ketone, *α*-terpineol and *trans*-2-hexenyl caproate were from Shanghai Yuanye Bio-Technology Co., Ltd. (Shanghai, China). Hexyl hexanoate was purchased from Shanghai Zhenzhun Bio-Technology Co., Ltd. (Shanghai, China). 4-methyl-3-penten-2-one was from Shanghai Yi’en Chemical Technology Co., Ltd. (Shanghai, China). Ethyl decanoate was from Shanghai ANPEL Scientific Instrument Co., Ltd. (Shanghai, China).

### Headspace solid-phase microextraction (HS-SPME)

A minor modification for HS-SPME was made according to the method in our previous paper^52^. Finely ground tea powders (2 g) were placed into a 20-mL sealed glass vial with 0.5 nmol ethyl decanoate as an internal standard. Polydimethylsiloxane-divinylbenzene (PDMS-DVB) SPME fiber was selected for volatile extraction following procedures reported in our previous work^52^.

### Gas chromatography-mass spectrometry (GC-MS)

The GC-MS system and the analytical conditions were the same as those in our previous paper^53^. GC separation was achieved on a Restek Rxi^®^-5Sil MS capillary column (30 m, inner diameter 0.25 mm and film thickness 0.25 µm). The oven temperature was programmed at 50 °C for 5 min, increased at 3 °C/min to 210 °C, then increased at 15 °C/min to 330 °C and kept for a 5 min final hold. The MS was operated in an electron impact (EI) mode with ionization energy of 70 eV and a mass scan range of 30–500 m/z. Samples were analyzed in triplicates. QC samples prepared by pooling aliquots of all samples were injected every ten runs throughout the analysis to monitor instrument fluctuations.

### Qualitative and quantitative analyses of volatile compounds

Raw data were processed with ChromaTOF software (v4.51.6, LECO) for spectral deconvolution and alignment. The following conditions were used: (1) signal-to-noise (S/N) ratio = 20; (2) maximum retention time difference = 2 s; (3) peak width = 5 s and (4) the mass spectral match score ≥700. The criteria used for the assignment of volatile metabolites were the same as in the previous work^52^. Relative quantification was made based on the peak area ratio of analytes to the internal standard.

### Multivariate and statistical analysis

Volatile data were auto-scaled, quantile-normalized and log10-transformed prior to hierarchical cluster analysis using the MetaboAnalyst 5.0 program (https://www.metaboanalyst.ca/). PCA and OPLS-DA analyses were conducted in Simca-P (v14.1, Umetrics, Umeå, Sweden). All data were presented as mean ± standard deviation (SD). The Wilcoxon rank sum test was applied to compare the differences in the volatile abundance between CRT and NCRT.

### Machine learning modeling

**Fig. 6:**
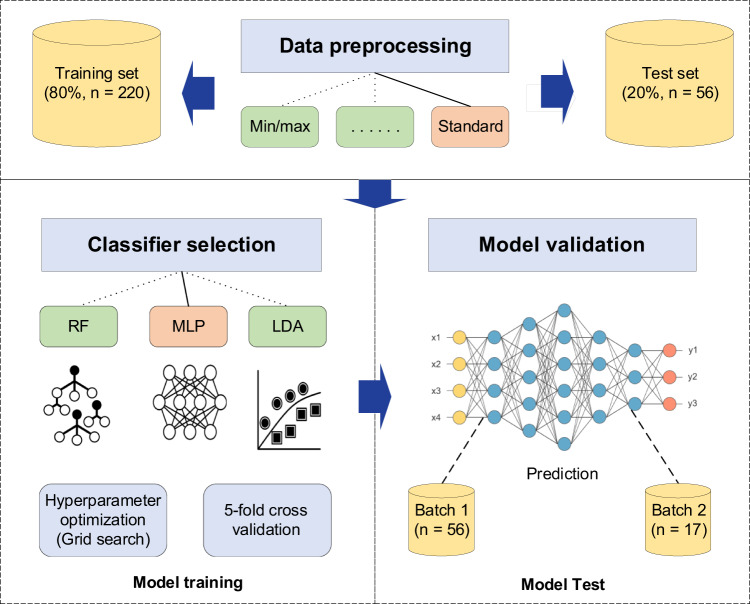
Diagram of the machine learning workflow. A flowchart for building tea geographic origin discrimination model is described, including data preprocessing, feature selection, hyperparameter optimization and model validation.

To generate a model for geographic origin discrimination of WRT, different ML algorithms, including multilayer perceptron (MLP), quadratic discriminant analysis (QDA), passive aggressive (PA), support vector machines (SVM), linear discriminant analysis (LDA), random forest (RF), stochastic gradient descent (SGD), gradient boosting (GB), adaboost (AB), k-nearest neighbors (KNN), linear support vector machines (LinearSVM), Bernoulli naive Bayes (BernoulliNB), extra tree (ET), Gaussian naive Bayes (GaussianNB) and decision tree (DT) were tested using Scikit-learn (v0.24.2) package in Python (v3.8.12) (Fig. 6)^54^. Metabolomics data were split into two parts: 80% for model training and 20% for validation. The training set was used to train the models with five-fold cross-validation. The full pipeline Bayesian hyperparameter optimization was performed to select the optimal ML method. We run 40 jobs in parallel on a machine with 80 cores for 24 h. For the resource limits, we set a time limit of 30 min for a single run and a memory limit for 100 GB^54,55^. Various classification metrics like accuracy, precision, recall, and AUC were calculated to evaluate the model performance.

### Reporting summary

Further information on research design is available in the Nature Research Reporting Summary linked to this article.

## Supplementary information

Supplementary Information

Reporting Summary

Supplementary Dataset 1

Supplementary Dataset 2

## Data Availability

The authors declare that all pertinent data that support this study have been included within the paper. Raw data will be made available by corresponding authors upon request.
