# Supplementary material for: Metabolomics integrated with machine learning to discriminate the geographic origin of Rougui Wuyi rock tea
Source: NPJ Sci Food. 2023 Mar 16;7:7. doi: 10.1038/s41538-023-00187-1 (PMC10020150; doi:10.1038/s41538-023-00187-1)
Supplement: Supplementary file 1 — Supplementary Information [file 41538_2023_187_MOESM1_ESM.pdf]

Supplementary Information

Metabolomics integrated with machine learning to discriminate the geographic origin of Rougui Wuyi rock tea

Yifei Peng[a,b#], Chao Zheng[b#], Shuang Guo[a,b], Fuquan Gao[a,b], Xiaxia Wang[b], Zhenghua Du[b], Feng Gao[c], Feng Su[c], Wenjing Zhang[c], Xueling Yu[c], Guoying Liu[d], Baoshun Liu[e], Chengjian Wu[f], Yun Sun[a], Zhenbiao Yang[b]*, Zhilong Hao[a]*, Xiaomin Yu[b]*

[a]College of Horticulture, Fujian Agriculture and Forestry University, Fuzhou 350002, China;

[b]FAFU-UCR Joint Center for Horticultural Biology and Metabolomics, Haixia Institute of Science and Technology, Fujian Agriculture and Forestry University, Fuzhou 350002, China;

[c]Fujian Farming Technology Extension Center, Fuzhou 350003, China;

[d]Wuyishan Institute of Agricultural Sciences, Wuyishan 354300, China;

[e]Wuyishan Tea Bureau, Wuyishan 354300, China;

[f]Fujian Vocational College of Agriculture, Fuzhou 350119, China.

[#] Y. P. and C.Z. contributed equally to this work.

***Correspondence:**

Dr. Zhenbiao Yang (yang@ucr.edu)

Dr. Zhilong Hao (haozhilong@126.com)

Dr. Xiaomin Yu (xmyu0616@fafu.edu.cn)

Table S1. Detailed sampling information for 333 Wuyi rock tea samples

Sample No.	Manufacture year	Declared region	Declared origin
**1**	2019	Mt. Wuyi Scenic Resort, Nanping City	CRT
**2**	2019	Mt. Wuyi Scenic Resort, Nanping City	CRT
**3**	2019	Mt. Wuyi Scenic Resort, Nanping City	CRT
**4**	2019	Mt. Wuyi Scenic Resort, Nanping City	CRT
**5**	2019	Mt. Wuyi Scenic Resort, Nanping City	CRT
**6**	2019	Mt. Wuyi Scenic Resort, Nanping City	CRT
**7**	2020	Mt. Wuyi Scenic Resort, Nanping City	CRT
**8**	2020	Mt. Wuyi Scenic Resort, Nanping City	CRT
**9**	2020	Mt. Wuyi Scenic Resort, Nanping City	CRT
**10**	2020	Mt. Wuyi Scenic Resort, Nanping City	CRT
**11**	2020	Mt. Wuyi Scenic Resort, Nanping City	CRT
**12**	2020	Mt. Wuyi Scenic Resort, Nanping City	CRT
**13**	2020	Mt. Wuyi Scenic Resort, Nanping City	CRT
**14**	2019	Mt. Wuyi Scenic Resort, Nanping City	CRT
**15**	2020	Mt. Wuyi Scenic Resort, Nanping City	CRT
**16**	2020	Mt. Wuyi Scenic Resort, Nanping City	CRT
**17**	2020	Mt. Wuyi Scenic Resort, Nanping City	CRT
**18**	2020	Mt. Wuyi Scenic Resort, Nanping City	CRT
**19**	2020	Mt. Wuyi Scenic Resort, Nanping City	CRT
**20**	2020	Mt. Wuyi Scenic Resort, Nanping City	CRT
**21**	2020	Mt. Wuyi Scenic Resort, Nanping City	CRT
**22**	2020	Mt. Wuyi Scenic Resort, Nanping City	CRT
**23**	2020	Mt. Wuyi Scenic Resort, Nanping City	CRT
**24**	2020	Mt. Wuyi Scenic Resort, Nanping City	CRT
**25**	2020	Mt. Wuyi Scenic Resort, Nanping City	CRT
**26**	2020	Mt. Wuyi Scenic Resort, Nanping City	CRT
**27**	2020	Mt. Wuyi Scenic Resort, Nanping City	CRT
**28**	2020	Mt. Wuyi Scenic Resort, Nanping City	CRT
**29**	2020	Mt. Wuyi Scenic Resort, Nanping City	CRT
**30**	2020	Mt. Wuyi Scenic Resort, Nanping City	CRT
**31**	2020	Mt. Wuyi Scenic Resort, Nanping City	CRT
**32**	2020	Mt. Wuyi Scenic Resort, Nanping City	CRT
**33**	2020	Mt. Wuyi Scenic Resort, Nanping City	CRT
**34**	2020	Mt. Wuyi Scenic Resort, Nanping City	CRT
**35**	2020	Mt. Wuyi Scenic Resort, Nanping City	CRT
**36**	2020	Mt. Wuyi Scenic Resort, Nanping City	CRT
**37**	2020	Mt. Wuyi Scenic Resort, Nanping City	CRT
**38**	2020	Mt. Wuyi Scenic Resort, Nanping City	CRT

**39**	2020	Mt. Wuyi Scenic Resort, Nanping City	CRT
**40**	2020	Mt. Wuyi Scenic Resort, Nanping City	CRT
**41**	2020	Mt. Wuyi Scenic Resort, Nanping City	CRT
**42**	2020	Mt. Wuyi Scenic Resort, Nanping City	CRT
**43**	2020	Mt. Wuyi Scenic Resort, Nanping City	CRT
**44**	2020	Mt. Wuyi Scenic Resort, Nanping City	CRT
**45**	2020	Mt. Wuyi Scenic Resort, Nanping City	CRT
**46**	2020	Mt. Wuyi Scenic Resort, Nanping City	CRT
**47**	2020	Mt. Wuyi Scenic Resort, Nanping City	CRT
**48**	2020	Mt. Wuyi Scenic Resort, Nanping City	CRT
**49**	2020	Mt. Wuyi Scenic Resort, Nanping City	CRT
**50**	2020	Mt. Wuyi Scenic Resort, Nanping City	CRT
**51**	2020	Mt. Wuyi Scenic Resort, Nanping City	CRT
**52**	2020	Mt. Wuyi Scenic Resort, Nanping City	CRT
**53**	2020	Mt. Wuyi Scenic Resort, Nanping City	CRT
**54**	2019	Mt. Wuyi Scenic Resort, Nanping City	CRT
**55**	2019	Mt. Wuyi Scenic Resort, Nanping City	CRT
**56**	2019	Mt. Wuyi Scenic Resort, Nanping City	CRT
**57**	2019	Mt. Wuyi Scenic Resort, Nanping City	CRT
**58**	2019	Mt. Wuyi Scenic Resort, Nanping City	CRT
**59**	2019	Mt. Wuyi Scenic Resort, Nanping City	CRT
**60**	2019	Mt. Wuyi Scenic Resort, Nanping City	CRT
**61**	2019	Mt. Wuyi Scenic Resort, Nanping City	CRT
**62**	2019	Mt. Wuyi Scenic Resort, Nanping City	CRT
**63**	2019	Mt. Wuyi Scenic Resort, Nanping City	CRT
**64**	2019	Mt. Wuyi Scenic Resort, Nanping City	CRT
**65**	2019	Mt. Wuyi Scenic Resort, Nanping City	CRT
**66**	2019	Mt. Wuyi Scenic Resort, Nanping City	CRT
**67**	2019	Mt. Wuyi Scenic Resort, Nanping City	CRT
**68**	2019	Mt. Wuyi Scenic Resort, Nanping City	CRT
**69**	2019	Mt. Wuyi Scenic Resort, Nanping City	CRT
**70**	2019	Mt. Wuyi Scenic Resort, Nanping City	CRT
**71**	2019	Mt. Wuyi Scenic Resort, Nanping City	CRT
**72**	2019	Mt. Wuyi Scenic Resort, Nanping City	CRT
**73**	2019	Mt. Wuyi Scenic Resort, Nanping City	CRT
**74**	2019	Mt. Wuyi Scenic Resort, Nanping City	CRT
**75**	2019	Mt. Wuyi Scenic Resort, Nanping City	CRT
**76**	2019	Mt. Wuyi Scenic Resort, Nanping City	CRT
**77**	2019	Mt. Wuyi Scenic Resort, Nanping City	CRT
**78**	2019	Mt. Wuyi Scenic Resort, Nanping City	CRT
**79**	2019	Mt. Wuyi Scenic Resort, Nanping City	CRT

**80**	2019	Mt. Wuyi Scenic Resort, Nanping City	CRT
**81**	2019	Mt. Wuyi Scenic Resort, Nanping City	CRT
**82**	2019	Mt. Wuyi Scenic Resort, Nanping City	CRT
**83**	2019	Mt. Wuyi Scenic Resort, Nanping City	CRT
**84**	2020	Mt. Wuyi Scenic Resort, Nanping City	CRT
**85**	2020	Mt. Wuyi Scenic Resort, Nanping City	CRT
**86**	2020	Mt. Wuyi Scenic Resort, Nanping City	CRT
**87**	2020	Mt. Wuyi Scenic Resort, Nanping City	CRT
**88**	2020	Mt. Wuyi Scenic Resort, Nanping City	CRT
**89**	2020	Mt. Wuyi Scenic Resort, Nanping City	CRT
**90**	2020	Mt. Wuyi Scenic Resort, Nanping City	CRT
**91**	2020	Mt. Wuyi Scenic Resort, Nanping City	CRT
**92**	2020	Mt. Wuyi Scenic Resort, Nanping City	CRT
**93**	2020	Mt. Wuyi Scenic Resort, Nanping City	CRT
**94**	2020	Mt. Wuyi Scenic Resort, Nanping City	CRT
**95**	2020	Mt. Wuyi Scenic Resort, Nanping City	CRT
**96**	2020	Mt. Wuyi Scenic Resort, Nanping City	CRT
**97**	2020	Mt. Wuyi Scenic Resort, Nanping City	CRT
**98**	2020	Mt. Wuyi Scenic Resort, Nanping City	CRT
**99**	2020	Mt. Wuyi Scenic Resort, Nanping City	CRT
**100**	2020	Mt. Wuyi Scenic Resort, Nanping City	CRT
**101**	2020	Mt. Wuyi Scenic Resort, Nanping City	CRT
**102**	2020	Mt. Wuyi Scenic Resort, Nanping City	CRT
**103**	2020	Mt. Wuyi Scenic Resort, Nanping City	CRT
**104**	2020	Mt. Wuyi Scenic Resort, Nanping City	CRT
**105**	2020	Mt. Wuyi Scenic Resort, Nanping City	CRT
**106**	2020	Mt. Wuyi Scenic Resort, Nanping City	CRT
**107**	2020	Mt. Wuyi Scenic Resort, Nanping City	CRT
**108**	2020	Mt. Wuyi Scenic Resort, Nanping City	CRT
**109**	2020	Mt. Wuyi Scenic Resort, Nanping City	CRT
**110**	2020	Mt. Wuyi Scenic Resort, Nanping City	CRT
**111**	2020	Mt. Wuyi Scenic Resort, Nanping City	CRT
**112**	2020	Mt. Wuyi Scenic Resort, Nanping City	CRT
**113**	2020	Mt. Wuyi Scenic Resort, Nanping City	CRT
**114**	2020	Mt. Wuyi Scenic Resort, Nanping City	CRT
**115**	2020	Mt. Wuyi Scenic Resort, Nanping City	CRT
**116**	2020	Mt. Wuyi Scenic Resort, Nanping City	CRT
**117**	2020	Mt. Wuyi Scenic Resort, Nanping City	CRT
**118**	2020	Mt. Wuyi Scenic Resort, Nanping City	CRT
**119**	2020	Mt. Wuyi Scenic Resort, Nanping City	CRT
**120**	2020	Mt. Wuyi Scenic Resort, Nanping City	CRT

**121**	2020	Mt. Wuyi Scenic Resort, Nanping City	CRT
**122**	2020	Mt. Wuyi Scenic Resort, Nanping City	CRT
**123**	2020	Mt. Wuyi Scenic Resort, Nanping City	CRT
**124**	2020	Mt. Wuyi Scenic Resort, Nanping City	CRT
**125**	2020	Mt. Wuyi Scenic Resort, Nanping City	CRT
**126**	2020	Mt. Wuyi Scenic Resort, Nanping City	CRT
**127**	2020	Mt. Wuyi Scenic Resort, Nanping City	CRT
**128**	2020	Mt. Wuyi Scenic Resort, Nanping City	CRT
**129**	2020	Mt. Wuyi Scenic Resort, Nanping City	CRT
**130**	2020	Mt. Wuyi Scenic Resort, Nanping City	CRT
**131**	2020	Mt. Wuyi Scenic Resort, Nanping City	CRT
**132**	2020	Mt. Wuyi Scenic Resort, Nanping City	CRT
**133**	2020	Mt. Wuyi Scenic Resort, Nanping City	CRT
**134**	2020	Mt. Wuyi Scenic Resort, Nanping City	CRT
**135**	2020	Mt. Wuyi Scenic Resort, Nanping City	CRT
**136**	2020	Mt. Wuyi Scenic Resort, Nanping City	CRT
**137**	2020	Mt. Wuyi Scenic Resort, Nanping City	CRT
**138**	2020	Mt. Wuyi Scenic Resort, Nanping City	CRT
**139**	2020	Mt. Wuyi Scenic Resort, Nanping City	CRT
**140**	2020	Mt. Wuyi Scenic Resort, Nanping City	CRT
**141**	2020	Mt. Wuyi Scenic Resort, Nanping City	CRT
**142**	2020	Mt. Wuyi Scenic Resort, Nanping City	CRT
**143**	2020	Mt. Wuyi Scenic Resort, Nanping City	CRT
**144**	2020	Mt. Wuyi Scenic Resort, Nanping City	CRT
**145**	2020	Mt. Wuyi Scenic Resort, Nanping City	CRT
**146**	2020	Mt. Wuyi Scenic Resort, Nanping City	CRT
**147**	2020	Mt. Wuyi Scenic Resort, Nanping City	CRT
**148**	2020	Mt. Wuyi Scenic Resort, Nanping City	CRT
**149**	2020	Mt. Wuyi Scenic Resort, Nanping City	CRT
**150**	2020	Mt. Wuyi Scenic Resort, Nanping City	CRT
**151**	2020	Mt. Wuyi Scenic Resort, Nanping City	CRT
**152**	2020	Mt. Wuyi Scenic Resort, Nanping City	CRT
**153**	2020	Mt. Wuyi Scenic Resort, Nanping City	CRT
**154**	2020	Mt. Wuyi Scenic Resort, Nanping City	CRT
**155**	2020	Mt. Wuyi Scenic Resort, Nanping City	CRT
**156**	2020	Mt. Wuyi Scenic Resort, Nanping City	CRT
**157**	2020	Mt. Wuyi Scenic Resort, Nanping City	CRT
**158**	2020	Mt. Wuyi Scenic Resort, Nanping City	CRT
**159**	2020	Mt. Wuyi Scenic Resort, Nanping City	CRT
**160**	2020	Mt. Wuyi Scenic Resort, Nanping City	CRT
**161**	2020	Mt. Wuyi Scenic Resort, Nanping City	CRT

**162**	2020	Mt. Wuyi Scenic Resort, Nanping City	CRT
**163**	2020	Mt. Wuyi Scenic Resort, Nanping City	CRT
**164**	2020	Mt. Wuyi Scenic Resort, Nanping City	CRT
**165**	2020	Mt. Wuyi Scenic Resort, Nanping City	CRT
**166**	2020	Mt. Wuyi Scenic Resort, Nanping City	CRT
**167**	2020	Mt. Wuyi Scenic Resort, Nanping City	CRT
**168**	2020	Mt. Wuyi Scenic Resort, Nanping City	CRT
**169**	2020	Mt. Wuyi Scenic Resort, Nanping City	CRT
**170**	2020	Mt. Wuyi Scenic Resort, Nanping City	CRT
**171**	2020	Mt. Wuyi Scenic Resort, Nanping City	CRT
**172**	2020	Mt. Wuyi Scenic Resort, Nanping City	CRT
**173**	2020	Mt. Wuyi Scenic Resort, Nanping City	CRT
**174**	2020	Mt. Wuyi Scenic Resort, Nanping City	CRT
**175**	2020	Yongan County, Sanming City	NCRT
**176**	2020	Taining County, Sanming City	NCRT
**177**	2020	Jianning County, Sanming City	NCRT
**178**	2020	Taining County, Sanming City	NCRT
**179**	2020	Taining County, Sanming City	NCRT
**180**	2020	Taining County, Sanming City	NCRT
**181**	2020	Taining County, Sanming City	NCRT
**182**	2020	Datian County, Sanming City	NCRT
**183**	2019	Wuyishan County, Nanping City	NCRT
**184**	2019	Wuyishan County, Nanping City	NCRT
**185**	2019	Wuyishan County, Nanping City	NCRT
**186**	2019	Wuyishan County, Nanping City	NCRT
**187**	2019	Wuyishan County, Nanping City	NCRT
**188**	2019	Wuyishan County, Nanping City	NCRT
**189**	2020	Wuyishan County, Nanping City	NCRT
**190**	2020	Wuyishan County, Nanping City	NCRT
**191**	2020	Wuyishan County, Nanping City	NCRT
**192**	2020	Wuyishan County, Nanping City	NCRT
**193**	2020	Wuyishan County, Nanping City	NCRT
**194**	2020	Wuyishan County, Nanping City	NCRT
**195**	2020	Wuyishan County, Nanping City	NCRT
**196**	2020	Wuyishan County, Nanping City	NCRT
**197**	2020	Wuyishan County, Nanping City	NCRT
**198**	2020	Wuyishan County, Nanping City	NCRT
**199**	2020	Wuyishan County, Nanping City	NCRT
**200**	2020	Wuyishan County, Nanping City	NCRT
**201**	2020	Wuyishan County, Nanping City	NCRT
**202**	2020	Wuyishan County, Nanping City	NCRT

**203**	2020	Wuyishan County, Nanping City	NCRT
**204**	2020	Wuyishan County, Nanping City	NCRT
**205**	2020	Wuyishan County, Nanping City	NCRT
**206**	2020	Wuyishan County, Nanping City	NCRT
**207**	2020	Wuyishan County, Nanping City	NCRT
**208**	2020	Taining County, Sanming City	NCRT
**209**	2020	Taining County, Sanming City	NCRT
**210**	2020	Pucheng County, Nanping City	NCRT
**211**	2020	Pucheng County, Nanping City	NCRT
**212**	2020	Wuyishan County, Nanping City	NCRT
**213**	2020	Wuyishan County, Nanping City	NCRT
**214**	2020	Wuyishan County, Nanping City	NCRT
**215**	2020	Wuyishan County, Nanping City	NCRT
**216**	2020	Wuyishan County, Nanping City	NCRT
**217**	2020	Wuyishan County, Nanping City	NCRT
**218**	2020	Wuyishan County, Nanping City	NCRT
**219**	2020	Wuyishan County, Nanping City	NCRT
**220**	2020	Wuyishan County, Nanping City	NCRT
**221**	2020	Wuyishan County, Nanping City	NCRT
**222**	2020	Jianyang County, Nanping City	NCRT
**223**	2020	Jianyang County, Nanping City	NCRT
**224**	2020	Wuyishan County, Nanping City	NCRT
**225**	2020	Wuyishan County, Nanping City	NCRT
**226**	2020	Wuyishan County, Nanping City	NCRT
**227**	2020	Wuyishan County, Nanping City	NCRT
**228**	2020	Taining County, Sanming City	NCRT
**229**	2020	Taining County, Sanming City	NCRT
**230**	2020	Taining County, Sanming City	NCRT
**231**	2020	Taining County, Sanming City	NCRT
**232**	2020	Taining County, Sanming City	NCRT
**233**	2020	Taining County, Sanming City	NCRT
**234**	2020	Taining County, Sanming City	NCRT
**235**	2020	Taining County, Sanming City	NCRT
**236**	2020	Taining County, Sanming City	NCRT
**237**	2020	Taining County, Sanming City	NCRT
**238**	2020	Taining County, Sanming City	NCRT
**239**	2020	Taining County, Sanming City	NCRT
**240**	2020	Taining County, Sanming City	NCRT
**241**	2020	Taining County, Sanming City	NCRT
**242**	2020	Taining County, Sanming City	NCRT
**243**	2020	Taining County, Sanming City	NCRT

**244**	2020	Jianou County, Nanping City	NCRT
**245**	2020	Jianou County, Nanping City	NCRT
**246**	2020	Jianou County, Nanping City	NCRT
**247**	2020	Jianou County, Nanping City	NCRT
**248**	2020	Jianou County, Nanping City	NCRT
**249**	2020	Jianou County, Nanping City	NCRT
**250**	2020	Jianou County, Nanping City	NCRT
**251**	2020	Jianou County, Nanping City	NCRT
**252**	2020	Jianou County, Nanping City	NCRT
**253**	2020	Jianou County, Nanping City	NCRT
**254**	2020	Jianou County, Nanping City	NCRT
**255**	2020	Jianou County, Nanping City	NCRT
**256**	2020	Jianou County, Nanping City	NCRT
**257**	2020	Jianou County, Nanping City	NCRT
**258**	2020	Jianou County, Nanping City	NCRT
**259**	2020	Jianou County, Nanping City	NCRT
**260**	2020	Jianou County, Nanping City	NCRT
**261**	2020	Jianou County, Nanping City	NCRT
**262**	2020	Jianou County, Nanping City	NCRT
**263**	2020	Jianou County, Nanping City	NCRT
**264**	2020	Jianou County, Nanping City	NCRT
**265**	2020	Jianou County, Nanping City	NCRT
**266**	2020	Jianou County, Nanping City	NCRT
**267**	2020	Jianou County, Nanping City	NCRT
**268**	2020	Jianou County, Nanping City	NCRT
**269**	2020	Jianou County, Nanping City	NCRT
**270**	2020	Jianou County, Nanping City	NCRT
**271**	2020	Jianou County, Nanping City	NCRT
**272**	2020	Jianou County, Nanping City	NCRT
**273**	2020	Jianou County, Nanping City	NCRT
**274**	2020	Jianou County, Nanping City	NCRT
**275**	2020	Jianou County, Nanping City	NCRT
**276**	2020	Jianou County, Nanping City	NCRT
**277**	2020	Jianou County, Nanping City	NCRT
**278**	2020	Jianou County, Nanping City	NCRT
**279**	2020	Jianou County, Nanping City	NCRT
**280**	2020	Jianou County, Nanping City	NCRT
**281**	2020	Jianou County, Nanping City	NCRT
**282**	2020	Jianou County, Nanping City	NCRT
**283**	2020	Jianou County, Nanping City	NCRT
**284**	2020	Jianou County, Nanping City	NCRT

**285**	2020	Jianou County, Nanping City	NCRT
**286**	2020	Jianou County, Nanping City	NCRT
**287**	2020	Jianou County, Nanping City	NCRT
**288**	2020	Jianou County, Nanping City	NCRT
**289**	2020	Jianou County, Nanping City	NCRT
**290**	2020	Wuyishan County, Nanping City	NCRT
**291**	2020	Wuyishan County, Nanping City	NCRT
**292**	2020	Wuyishan County, Nanping City	NCRT
**293**	2020	Wuyishan County, Nanping City	NCRT
**294**	2020	Wuyishan County, Nanping City	NCRT
**295**	2020	Wuyishan County, Nanping City	NCRT
**296**	2020	Wuyishan County, Nanping City	NCRT
**297**	2020	Wuyishan County, Nanping City	NCRT
**298**	2020	Wuyishan County, Nanping City	NCRT
**299**	2020	Wuyishan County, Nanping City	NCRT
**300**	2020	Wuyishan County, Nanping City	NCRT
**301**	2020	Wuyishan County, Nanping City	NCRT
**302**	2020	Wuyishan County, Nanping City	NCRT
**303**	2020	Wuyishan County, Nanping City	NCRT
**304**	2020	Wuyishan County, Nanping City	NCRT
**305**	2020	Wuyishan County, Nanping City	NCRT
**306**	2020	Wuyishan County, Nanping City	NCRT
**307**	2020	Wuyishan County, Nanping City	NCRT
**308**	2020	Wuyishan County, Nanping City	NCRT
**309**	2020	Wuyishan County, Nanping City	NCRT
**310**	2020	Wuyishan County, Nanping City	NCRT
**311**	2020	Wuyishan County, Nanping City	NCRT
**312**	2020	Wuyishan County, Nanping City	NCRT
**313**	2020	Wuyishan County, Nanping City	NCRT
**314**	2020	Wuyishan County, Nanping City	NCRT
**315**	2020	Wuyishan County, Nanping City	NCRT
**316**	2020	Wuyishan County, Nanping City	NCRT
**317**	2020	Wuyishan County, Nanping City	NCRT
**318**	2020	Wuyishan County, Nanping City	NCRT
**319**	2020	Wuyishan County, Nanping City	NCRT
**320**	2020	Wuyishan County, Nanping City	NCRT
**321**	2020	Wuyishan County, Nanping City	NCRT
**322**	2020	Wuyishan County, Nanping City	NCRT
**323**	2020	Wuyishan County, Nanping City	NCRT
**324**	2020	Wuyishan County, Nanping City	NCRT
**325**	2020	Wuyishan County, Nanping City	NCRT

**326**	2020	Wuyishan County, Nanping City	NCRT
**327**	2020	Wuyishan County, Nanping City	NCRT
**328**	2020	Wuyishan County, Nanping City	NCRT
**329**	2020	Wuyishan County, Nanping City	NCRT
**330**	2020	Wuyishan County, Nanping City	NCRT
**331**	2020	Wuyishan County, Nanping City	NCRT
**332**	2020	Wuyishan County, Nanping City	NCRT
**333**	2020	Wuyishan County, Nanping City	NCRT

Table S2. Tea volatiles used for multivariate analysis

No.	Compound	CAS No.	RT (min)	RI_{exp}	RI_{lit}	Identification method	Used for modeling
**1**	Ethyl isopropyl ketone	565-69-5	3.4	NA	745	MS, Std	Yes
**2**	*β*-hydroxybutyraldehyde	107-89-1	3.4	NA	NA	MS	
**3**	3-methyl-2-butenal	107-86-8	3.4	NA	NA	MS	
**4**	Pentyl alcohol	71-41-0	3.7	NA	756-766	MS, Std	Yes
**5**	Toluene	108-88-3	3.7	NA	767-787	MS	
**6**	*cis*-2-penten-1-ol	1576-95-0	3.7	NA	753-768	MS, Std	Yes
**7**	Unknown1	NA	3.8	NA	NA	ND	Yes
**8**	Unknown2	NA	4.0	NA	NA	ND	
**9**	Unknown3	NA	4.1	NA	NA	ND	Yes
**10**	4-methyl-3-pentene-2-one	141-79-7	4.3	NA	792-800	MS, Std	Yes
**11**	(2,3,3-trimethyloxiranyl)methanol	110933-26-1	4.4	NA	NA	MS	
**12**	Hexanal	66-25-1	4.4	800	800	MS, RI	Yes
**13**	Dihydro-2-methyl-3(2H)-furanone	3188-00-9	4.6	805	804	MS, RI	Yes
**14**	Unknown4	NA	4.6	805	NA	ND	
**15**	1-ethyl-1H-pyrrole	617-92-5	4.7	808	820	MS, RI	Yes
**16**	Unknown5	NA	4.8	810	NA	ND	
**17**	Hexamethylcyclotrisiloxane	541-05-9	4.8	812	NA	MS	Yes
**18**	Methylpyrazine	109-08-0	5.1	819	827	MS, RI	Yes
**19**	Furfuryl methyl ether	13679-46-4	5.2	821	NA	MS	
**20**	Unknown6	NA	5.3	824	NA	ND	Yes
**21**	Furfural	98-01-1	5.3	825	830	MS, RI	Yes
**22**	Unknown7	NA	5.5	831	NA	ND	
**23**	2-ethyl-3-methylbutanal	26254-92-2	5.5	831	NA	MS	Yes
**24**	3-methyl-hepta-1,6-dien-3-ol	34780-69-3	5.8	839	NA	MS	
**25**	3-bromopentane	1809-10-5	5.8	840	NA	MS	Yes
**26**	2,4,5-trimethyloxazole	20662-84-4	5.9	843	838-857	MS, RI	
**27**	Furfuryl alcohol	98-00-0	6.1	847	851	MS, RI	Yes
**28**	3-hexen-1-ol	544-12-7	6.2	849	845-856	MS, RI	Yes
**29**	Unknown8	NA	6.3	852	NA	ND	Yes
**30**	Unknown9	NA	6.4	856	NA	ND	
**31**	Unknown10	NA	6.5	858	NA	ND	
**32**	Acetoxyacetone	592-20-1	6.6	859	861-883	MS, RI	
**33**	(*E*)-2-hexen-1-ol	928-95-0	6.6	860	861-887	MS, RI, Std	
**34**	*p*-xylene	106-42-3	6.7	863	861-877	MS, RI, Std	
**35**	1-hexanol	111-27-3	6.7	864	865	MS, RI, Std	
**36**	Unknown11	NA	7.1	875	NA	ND	
**37**	2,6-dimethyl-1,5-heptadiene	6709-39-3	7.2	878	NA	MS	
**38**	Unknown12	NA	7.3	880	NA	ND	

**39**	2-heptanone	110-43-0	7.5	885	889	MS, RI, Std	Yes
**40**	Unknown13	NA	7.5	887	NA	ND	Yes
**41**	Unknown14	NA	7.6	887	NA	ND	
**42**	*β*-methylcrotonic acid	541-47-9	7.6	890	NA	MS	
**43**	Unknown15	NA	7.7	890	NA	ND	Yes
**44**	*cis*-4-hepten-1-al	6728-31-0	7.9	896	898	MS, RI	Yes
**45**	3-methylcyclopentyl acetate	24070-70-0	7.9	898	NA	MS	
**46**	Heptanal	111-71-7	8.0	899	903-896	MS, RI, Std	Yes
**47**	2-furanone	20825-71-2	8.2	904	913	MS, RI	Yes
**48**	2-acetylfuran	1192-62-7	8.3	905	910	MS, RI	
**49**	Butyrolactone	96-48-0	8.3	905	908-925	MS, RI	
**50**	Ethenyl formate	692-45-5	8.3	907	NA	MS	
**51**	2,5-dimethylpyrazine	123-32-0	8.4	908	915	MS, RI, Std	Yes
**52**	Unknown16	NA	8.5	910	NA	ND	
**53**	Ethylpyrazine	13925-00-3	8.5	911	910	MS, RI	Yes
**54**	2,3-dimethylpyrazine	5910-89-4	8.7	913	915	MS, RI	Yes
**55**	Hexanoic acid, methyl ester	106-70-7	9.0	921	910-936	MS, RI, Std	Yes
**56**	Methyl (*Z*)-3-hexenoate	13894-62-7	9.3	927	NA	MS	
**57**	*β*-angelica lactone	591-11-7	9.5	931	946	MS, RI	Yes
**58**	Unknown17	NA	10.1	943	NA	ND	Yes
**59**	5,5-dimethyl-2(5H)-furanone	20019-64-1	10.1	945	952	MS, RI	Yes
**60**	7,7-dichloro-2-heptanone	66241-43-8	10.4	950	NA	MS	
**61**	(*E*)-2-heptenal	18829-55-5	10.5	953	954	MS, RI	Yes
**62**	2-formyl-5-methylfuran	620-02-0	10.7	956	946-978	MS, RI	Yes
**63**	Unknown18	NA	11.0	962	NA	ND	
**64**	Unknown19	NA	11.0	963	NA	ND	
**65**	1-heptanol	111-70-6	11.3	969	970	MS, RI, Std	Yes
**66**	Methyl 2-furoate	611-13-2	11.3	969	NA	MS	Yes
**67**	3,5,5-trimethyl-2-hexene	26456-76-8	11.4	971	NA	MS	
**68**	Unknown20	NA	11.4	972	NA	ND	
**69**	1-octen-3-one	4312-99-6	11.5	974	962-982	MS, RI	
**70**	4,5-dimethyl-2-isopropyloxazole	19519-45-0	11.6	975	NA	MS	
**71**	1-octen-3-ol	3391-86-4	11.7	978	958-981	MS, RI, Std	Yes
**72**	6-methyl-5-heptene-2-one	110-93-0	11.9	982	986	MS, RI, Std	Yes
**73**	2-methyl 6-methylene 2-octene	10054-09-8	11.9	982	NA	MS	
**74**	Unknown21	NA	11.9	983	NA	ND	
**75**	Unknown22	NA	12.1	986	NA	ND	
**76**	*β*-pinene	127-91-3	12.1	987	981	MS, RI, Std	
**77**	2-pentylfuran	3777-69-3	12.2	988	972-996	MS, RI	Yes
**78**	1,2,3-trimethylbenzene	526-73-8	12.2	989	996	MS, RI	Yes
**79**	2-ethyl-3-methylpyrazine	15707-23-0	12.5	994	995	MS, RI	

**80**	2,3,5-trimethylpyrazine	14667-55-1	12.6	997	989-1017	MS, RI	
**81**	*trans*-2,6-dimethyl-2-6-octadiene	2609-23-6	12.6	998	976-990	MS, RI	
**82**	2-ethyl-6-methylpyrazine	13925-03-6	12.7	999	982-1010	MS, RI	
**83**	Octanal	124-13-0	12.8	1001	1001	MS, RI, Std	Yes
**84**	Hexanoic acid	142-62-1	13.0	1005	952-1038	MS, RI	Yes
**85**	Unknown23	NA	13.0	1005	NA	ND	
**86**	Unknown24	NA	13.0	1006	NA	ND	
**87**	(*E*,*E*)-2,4-heptadienal	4313-03-5	13.2	1009	1009	MS, RI	Yes
**88**	2-formyl pyrrole	1003-29-8	13.4	1014	1031	MS, RI	Yes
**89**	5-methyl-5,6-dihydro-2(1H)-pyridinone	NA	13.4	1014	NA	MS	
**90**	Mesitylene	108-67-8	13.5	1016	957-1003	MS, RI	
**91**	Dehydrosabinaketone	36262-12-1	13.6	1017	1116-1119	MS, RI	Yes
**92**	(*E*)-3-hexenoic acid	1577-18-0	13.7	1020	983-1023	MS, RI	Yes
**93**	Isovaleramide	541-46-8	13.8	1020	1018	MS, RI	
**94**	*o*-cymene	527-84-4	13.8	1021	1020	MS, RI, Std	Yes
**95**	Limonene	138-86-3	14.1	1026	1027	MS, RI, Std	Yes
**96**	3,4-dimethylfuran-2,5-dione	766-39-2	14.1	1026	1038	MS, RI	
**97**	(*E*)-4-oxohex-2-enal	2492-43-5	14.2	1029	958-976	MS, RI	
**98**	2-acetyl-5-methylfuran	1193-79-9	14.2	1029	1037	MS, RI, Std	Yes
**99**	Butanedioic acid, dimethyl ester	106-65-0	14.3	1030	1021-1035	MS, RI	Yes
**100**	1,1,3-trimethyl-2-cyclohexanone	2408-37-9	14.3	1031	1027-1047	MS, RI	Yes
**101**	Benzyl alcohol	100-51-6	14.4	1032	1030-1052	MS, RI, Std	Yes
**102**	5-methyl-5-vinyldihydrofuran-2(3H)-one	1073-11-6	14.4	1032	1046-1071	MS, RI	Yes
**103**	*trans*-β-Ocimene	3779-61-1	14.5	1035	1046	MS, RI	Yes
**104**	3-octen-2-one	1669-44-9	14.6	1036	1036-1040	MS, RI	
**105**	Pantolactone	599-04-2	14.7	1038	NA	MS	Yes
**106**	Benzeneacetaldehyde	122-78-1	14.7	1039	1041	MS, RI, Std	Yes
**107**	1-ethyl-1H-pyrrole-2-carboxaldehyde	2167-14-8	14.9	1043	1046	MS, RI	Yes
**108**	β-ocimene	13877-91-3	15.0	1045	1023-1050	MS, RI, Std	Yes
**109**	γ-caprolactone	695-06-7	15.1	1047	1047-1068	MS, RI	Yes
**110**	(*E*)-6-methylhept-4-en-1-ol	855901-81-4	15.3	1051	1020	MS, RI	
**111**	Unknown25	NA	15.5	1054	NA	ND	
**112**	3-methyl-2-cyclohexen-1-one	1193-18-6	15.5	1055	1010-1039	MS, RI	
**113**	(*E*)-2-Octenal	2548-87-0	15.5	1055	1058	MS, RI, Std	Yes
**114**	Methylthiohexanoate	2432-77-1	15.6	1057	1063	MS, RI	
**115**	2-furoylacetonitrile	31909-58-7	15.7	1059	NA	MS	Yes
**116**	Unknown26	NA	15.7	1060	NA	ND	

**117**	Acetophenone	98-86-2	15.8	1060	1065	MS, RI	Yes
**118**	4,5-dimethyl-2-isobutyloxazole	26131-91-9	15.8	1061	1044	MS, RI	
**119**	Dihydro-3-methylene-5-methyl-2-furanone	62873-16-9	16.0	1064	1074.5	MS, RI	
**120**	2-acetylpyrrole	1072-83-9	16.0	1064	1064	MS, RI, Std	Yes
**121**	(*E*,*E*)-3,5-octadien-2-one	30086-02-3	16.1	1067	1068-1107	MS, RI	Yes
**122**	*cis*-linalool 3,6-oxide	5989-33-3	16.2	1068	1070-1087	MS, RI	Yes
**123**	2-isopropylidene-α-methyl-cyclopropanemethanol	NA	16.2	1069	NA	MS	
**124**	1-octanol	111-87-5	16.3	1071	1063-1079	MS, RI, Std	Yes
**125**	3-ethyl-2,5-dimethylpyrazine	13360-65-1	16.4	1073	1069	MS, RI	Yes
**126**	Unknown27	NA	16.5	1074	NA	ND	
**127**	Unknown28	NA	16.6	1076	NA	ND	Yes
**128**	Unknown29	NA	16.8	1079	NA	ND	
**129**	Unknown30	NA	16.8	1080	NA	ND	
**130**	Unknown31	NA	16.8	1081	NA	ND	
**131**	4-methyl-3-(1-methylethylidene)-1-cyclohexene	NA	16.9	1082	1076	MS, RI	
**132**	*trans*-linalool oxide (furanoid)	34995-77-2	17.0	1084	1065-1098	MS, RI	Yes
**133**	Unknown32	NA	17.1	1087	NA	ND	
**134**	2-nonanone	821-55-6	17.2	1089	1052-1102	MS, RI, Std	Yes
**135**	Unknown33	NA	17.3	1090	NA	ND	
**136**	3,5-octadien-2-one	38284-27-4	17.3	1090	1068-1198	MS, RI	
**137**	1,5-dimethyl-1,4-cyclohexadiene	4190-06-1	17.4	1092	NA	MS	
**138**	Unknown34	NA	17.5	1095	NA	ND	Yes
**139**	Linalool	78-70-6	17.7	1098	1080-1110	MS, RI, Std	Yes
**140**	Unknown35	NA	17.7	1099	NA	ND	
**141**	6-methyl-3,5-heptadiene-2-one	1604-28-0	17.8	1100	1088-1105	MS, RI	
**142**	Hotrienol	29957-43-5	17.9	1102	1107	MS, RI	Yes
**143**	Nonanal	124-19-6	17.9	1103	1098-1104	MS, RI, Std	
**144**	Unknown36	NA	18.0	1105	NA	ND	
**145**	Maltol	118-71-8	18.1	1106	1060-1110	MS, RI	
**146**	Unknown37	NA	18.1	1107	NA	ND	
**147**	Phenylethyl alcohol	60-12-8	18.2	1109	1110-1141	MS, RI, Std	Yes
**148**	(*E*)-4,8-dimethylnona-1,3,7-triene	19945-61-0	18.4	1112	1113	MS, RI	Yes
**149**	6-ethyl-2-methyl-octane	62016-19-7	18.4	1112	NA	MS	
**150**	Methyl pyrrole-2-carboxylate	1193-62-0	18.5	1115	NA	MS	
**151**	Isophorone	78-59-1	18.6	1116	1118	MS, RI, Std	Yes
**152**	Unknown38	NA	18.8	1121	NA	ND	

**153**	Octanoic acid, methyl ester	111-11-5	18.9	1122	1105-1130	MS, RI	Yes
**154**	Unknown39	NA	19.0	1125	NA	ND	Yes
**155**	1,3,8-*p*-menthatriene	18368-95-1	19.1	1127	1118.7	MS, RI	
**156**	*N*-ethylsuccinimide	2314-78-5	19.2	1129	NA	MS	Yes
**157**	Unknown40	NA	19.3	1131	NA	ND	
**158**	Benzyl nitrile	140-29-4	19.4	1133	1135-1160	MS, RI, Std	Yes
**159**	Unknown41	NA	19.5	1134	NA	ND	
**160**	Unknown42	NA	19.6	1136	NA	ND	
**161**	(*R*,*S*)-5-ethyl-6-methyl-3E-hepten-2-one	57283-79-1	19.7	1139	NA	MS	
**162**	2,3-dihydro-3,5-dihydroxy-6-methyl-4h-pyran-4-one	28564-83-2	19.7	1139	1130-1151	MS, RI	Yes
**163**	Unknown43	NA	19.8	1140	NA	ND	
**164**	Cyclomethicone	541-02-6	19.8	1141	1134-1215	MS, RI	
**165**	Unknown44	NA	19.9	1143	NA	ND	Yes
**166**	1,2,3,5-tetramethylbenzene	527-53-7	20.0	1144	1097-1149	MS, RI	Yes
**167**	Unknown45	NA	20.0	1146	NA	ND	
**168**	1,4-dimethyl-4-acetylcyclohexene	43219-68-7	20.2	1148	1145-1152	MS, RI	
**169**	Nerol oxide	1786-08-9	20.2	1149	1144-1172	MS, RI	
**170**	(*E*,*Z*)-2,6-nonadienal	557-48-2	20.2	1149	1145	MS, RI	Yes
**171**	Unknown46	NA	20.3	1151	NA	ND	
**172**	Methyl 1,5-dimethyl-2-pyrrolecarboxylate	73476-31-0	20.5	1154	NA	MS	
**173**	2-nonenal	18829-56-6	20.6	1157	1139-1171	MS, RI	
**174**	Acetic acid, phenylmethyl ester	140-11-4	20.7	1158	1161-1170	MS, RI, Std	
**175**	Benzoyl isothiocyanate	532-55-8	20.7	1159	NA	MS	Yes
**176**	Unknown47	NA	20.8	1161	NA	ND	
**177**	Unknown48	NA	21.0	1166	NA	ND	Yes
**178**	Unknown49	NA	21.1	1166	NA	ND	
**179**	(*3R*,*6S*)-2,2,6-trimethyl-6-vinyltetrahydro-2H-pyran-3-ol	39028-58-5	21.1	1167	1162-1173	MS, RI	Yes
**180**	3-methyl-undecane	1002-43-3	21.2	1170	1169-1173	MS, RI	
**181**	Methyl benzeneacetate	101-41-7	21.3	1171	1177	MS, RI	
**182**	2,2,6-trimethyl-6-vinyltetrahydro-2H-pyran-3-ol	14049-11-7	21.3	1172	1163	MS, RI	
**183**	Unknown50	NA	21.4	1173	NA	ND	
**184**	Naphthalene	91-20-3	21.6	1176	1171-1192	MS, RI, Std	
**185**	Unknown51	NA	21.6	1178	NA	ND	
**186**	*p*-acetyltoluene	122-00-9	21.7	1179	1178-1183	MS, RI	
**187**	*cis*-butyric acid, 3-hexenyl ester	16491-36-4	21.9	1183	1179-1189	MS, RI, Std	Yes
**188**	2-methyl-3(2-furyl)acrolein	874-66-8	22.0	1185	NA	MS	

**189**	Unknown52	NA	22.0	1186	NA	ND	
**190**	Methyl salicylate	119-36-8	22.0	1186	1188-1234	MS, RI, Std	Yes
**191**	Unknown53	NA	22.1	1187	NA	ND	
**192**	2,6-dimethyl-3,7-octadiene-2,6-diol	13741-21-4	22.2	1189	1173-1191	MS, RI	
**193**	Butanoic acid, hexyl ester	2639-63-6	22.2	1190	1190-1195	MS, RI	Yes
**194**	α-terpineol	98-55-5	22.3	1191	1150-1200	MS, RI, Std	Yes
**195**	*trans*-2-hexenyl butyrate	53398-83-7	22.3	1192	1191	MS, RI	
**196**	Safranal	116-26-7	22.4	1193	1197-1212	MS, RI	Yes
**197**	Unknown54	NA	22.5	1195	NA	ND	
**198**	5-ethylundecane	17453-94-0	22.7	1199	1232	MS, RI	
**199**	Decanal	112-31-2	22.9	1203	1161-1228	MS, RI, Std	
**200**	Unknown55	NA	22.9	1204	NA	ND	
**201**	2,4-*trans*,*trans*-nonadienal	5910-87-2	23.2	1211	1210	MS, RI	Yes
**202**	Unknown56	NA	23.3	1212	NA	ND	
**203**	β-cyclocitral	432-25-7	23.3	1213	1219-1224	MS, RI	Yes
**204**	Isopentyloxyethyl acetate	204652-53-9	23.4	1215	1176	MS, RI	Yes
**205**	Unknown57	NA	23.5	1216	NA	ND	
**206**	Unknown58	NA	23.6	1218	NA	ND	
**207**	Unknown59	NA	23.6	1219	NA	ND	Yes
**208**	2,8-dimethylundecane	17301-25-6	23.7	1221	1218-1221	MS, RI	
**209**	*cis*-geraniol	106-25-2	23.7	1222	1220-1239	MS, RI	
**210**	Unknown60	NA	23.8	1224	NA	ND	
**211**	*cis*-3-hexenyl-α-methylbutyrate	53398-85-9	24.0	1228	1233	MS, RI	Yes
**212**	Ethylmethylmaleimide	20189-42-8	24.1	1230	1234-1280	MS, RI	Yes
**213**	Hexyl 2-methylbutyrate	10032-15-2	24.3	1234	1236	MS, RI, Std	Yes
**214**	*trans*-2-hexenyl isovalerate	68698-59-9	24.3	1234	1244	MS, RI	Yes
**215**	3,4-epoxy-3-ethyl-2-butanone	17257-82-8	24.4	1236	NA	MS	Yes
**216**	Unknown61	NA	24.6	1239	NA	ND	
**217**	Unknown62	NA	24.6	1241	NA	ND	
**218**	Unknown63	NA	24.8	1245	NA	ND	
**219**	Unknown64	NA	24.8	1245	NA	ND	
**220**	Unknown65	NA	25.0	1248	NA	ND	Yes
**221**	Geraniol	106-24-1	25.0	1249	1255-1277	MS, RI	Yes
**222**	Acetic acid, 2-phenylethyl ester	103-45-7	25.0	1249	1226-1279	MS, RI	Yes
**223**	2-butyl-1-octanol	3913-02-8	25.1	1252	1277	MS, RI	
**224**	Unknown66	NA	25.2	1253	NA	ND	Yes
**225**	Hexanoic anhydride	2051-49-2	25.3	1254	NA	MS	
**226**	Methylvinylmaleimide	21494-57-5	25.3	1256	1261	MS, RI	
**227**	Unknown67	NA	25.3	1256	NA	ND	
**228**	Benzeneacetic acid	103-82-2	25.4	1257	1246-1279	MS, RI	

**229**	(*Z*)-2-Decenal	2497-25-8	25.4	1258	1249-1254	MS, RI	Yes
**230**	3,7-dimethylundecane	17301-29-0	25.6	1261	1221-1222	MS, RI	
**231**	α-ethylidene-phenylacetaldehyde	4411-89-6	25.6	1262	1273	MS, RI	
**232**	9-oxabicyclo[6.1.0]non-6-en-2-one	NA	25.7	1264	NA	MS	
**233**	Unknown68	NA	25.8	1267	NA	ND	
**234**	2-methyl-1-nonen-3-yne	70058-00-3	25.9	1267	NA	MS	Yes
**235**	Unknown69	NA	25.9	1269	NA	ND	Yes
**236**	2,3,6,7-tetramethyloctane	52670-34-5	26.1	1273	NA	MS	Yes
**237**	Hexanoic acid, 1-cyclopentylethyl ester	959244-69-0	26.2	1275	NA	MS	
**238**	(*E*)-4-decen-6-yne	13343-77-6	26.3	1276	1244	MS, RI	
**239**	2-azido-1-(pyridine-3-yl)ethanol	NA	26.3	1277	NA	MS	
**240**	*cis*-3-hexenyl isovalerate	35154-45-1	26.4	1280	1243	MS, RI	Yes
**241**	2,6,11-trimethyldodecane	31295-56-4	26.5	1282	1275	MS, RI	Yes
**242**	Unknown70	NA	26.7	1284	NA	ND	
**243**	Indole	120-72-9	26.8	1286	1292	MS, RI, Std	Yes
**244**	2,7,10-trimethyldodecane	74645-98-0	26.8	1287	NA	MS	
**245**	Unknown71	NA	26.9	1289	NA	ND	
**246**	2,4-decadienal	2363-88-4	26.9	1290	1293-1340	MS, RI	
**247**	1-nitro-2-phenylethane	6125-24-2	27.0	1291	1300-1305	MS, RI	Yes
**248**	Unknown72	NA	27.1	1294	NA	ND	Yes
**249**	2-(3-methylbutyl)-3,5-dimethylpyrazine	111150-30-2	27.2	1296	NA	MS	
**250**	2,4,6,8-tetramethyl-1-undecene	59920-26-2	27.3	1298	1275-1295	MS, RI	
**251**	Unknown73	NA	27.4	1300	NA	ND	Yes
**252**	Unknown74	NA	27.4	1301	NA	ND	
**253**	2-methoxy-4-vinylphenol	7786-61-0	27.6	1304	1292-1324	MS, RI	
**254**	Unknown75	NA	27.6	1305	NA	ND	
**255**	Unknown76	NA	27.7	1306	NA	ND	
**256**	Dodecamethylcyclohexasiloxane	540-97-6	27.7	1307	1319-1349	MS, RI	
**257**	Unknown77	NA	27.7	1307	NA	ND	
**258**	Isomyocorene	6876-07-9	28.0	1313	NA	MS	
**259**	4,6,8-trimethyl-1-nonene	54410-98-9	28.0	1314	NA	MS	Yes
**260**	(*E*)-methyl geranate	1189-09-9	28.1	1317	1315	MS, RI	Yes
**261**	4,6-dimethyldodecane	61141-72-8	28.2	1319	1325	MS, RI	Yes
**262**	Unknown78	NA	28.5	1325	NA	ND	
**263**	Unknown79	NA	28.7	1329	NA	ND	
**264**	5-isopropylfuran-2(5H)-one	56767-19-2	28.8	1332	1311	MS, RI	
**265**	2,3,5,8-tetramethyldecane	192823-15-7	29.0	1337	1318	MS, RI	Yes
**266**	Unknown80	NA	29.1	1339	NA	ND	

**267**	Unknown81	NA	29.1	1339	NA	ND	
**268**	Triacetin	102-76-1	29.2	1340	1339-1350	MS, RI	
**269**	Unknown82	NA	29.3	1343	NA	ND	
**270**	Unknown83	NA	29.3	1344	NA	ND	
**271**	1, 1, 5-trimethyl-1, 2-dihydronaphthalene	NA	29.4	1346	NA	MS	
**272**	Unknown84	NA	29.5	1347	NA	ND	Yes
**273**	1,1,4,5-tetramethylindane	16204-57-2	29.5	1348	1355	MS, RI	
**274**	Unknown85	NA	29.6	1350	NA	ND	
**275**	*γ*-n-amylbutyrolactone	104-61-0	29.7	1353	1344-1370	MS, RI	Yes
**276**	Nerolic acid	4613-38-1	30.0	1359	1340	MS, RI	
**277**	Unknown86	NA	30.1	1362	NA	ND	
**278**	3-methyltridecane	6418-41-3	30.4	1368	1371-1375	MS, RI	Yes
**279**	Unknown87	NA	30.4	1369	NA	ND	Yes
**280**	(+)-isopinocampheol	24041-60-9	30.5	1370	NA	MS	
**281**	4-methyltridecane	26730-12-1	30.6	1373	1355-1360	MS, RI	
**282**	*cis*-3-hexenyl hexanoate	31501-11-8	30.8	1376	1379-1386	MS, RI, Std	Yes
**283**	Unknown88	NA	30.9	1379	NA	ND	
**284**	*cis*-3-hexenyl *cis*-3-hexenoate	61444-38-0	30.9	1380	1388	MS, RI	Yes
**285**	Hexyl hexanoate	6378-65-0	31.0	1382	1384	MS, RI, Std	Yes
**286**	*trans*-2-hexenyl caproate	53398-86-0	31.1	1384	1391	MS, RI, Std	Yes
**287**	Hexanoic acid, cyclohexyl ester	6243-10-3	31.1	1385	1398-1411	MS, RI	Yes
**288**	*cis*-jasmone	488-10-8	31.2	1386	1392-1404	MS, RI	Yes
**289**	Phenethyl isobutyrate	103-48-0	31.2	1387	1395-1403	MS, RI	Yes
**290**	Unknown89	NA	31.3	1389	NA	ND	
**291**	Unknown90	NA	31.5	1394	NA	ND	
**292**	Tetradecane	629-59-4	31.7	1397	NA	MS	
**293**	2-dodecanone	6175-49-1	31.7	1398	1348-1410	MS, RI	
**294**	Unknown91	NA	31.8	1399	NA	ND	Yes
**295**	2,4-pentadienoic acid, 1-cyclopenten-3-on-1-yl ester	NA	31.9	1402	NA	MS	Yes
**296**	*Z*-2-dodecenol	69064-36-4	32.0	1404	NA	MS	
**297**	Unknown92	NA	32.1	1408	NA	ND	
**298**	Unknown93	NA	32.2	1410	NA	ND	
**299**	Unknown94	NA	32.3	1413	NA	ND	
**300**	*α*-ionone	127-41-3	32.4	1415	1418-1456	MS, RI	Yes
**301**	Unknown95	NA	32.6	1420	NA	ND	
**302**	*β*-elemene	29873-99-2	32.7	1422	1362-1408	MS, RI	
**303**	Coumarin	91-64-5	32.8	1424	1429-1456	MS, RI	Yes
**304**	Unknown96	NA	32.9	1427	NA	ND	
**305**	Unknown97	NA	33.0	1428	NA	ND	
**306**	Unknown98	NA	33.1	1430	NA	ND	

**307**	β-phenylethyl butyrate	103-52-6	33.1	1432	1439-1447	MS, RI, Std	Yes
**308**	Unknown99	NA	33.2	1433	NA	ND	
**309**	Unknown100	NA	33.2	1434	NA	ND	
**310**	(*E*)-5,6-epoxy-β-ionone	23267-57-4	33.2	1434	1455	MS, RI	Yes
**311**	Isoeugenol	97-54-1	33.5	1440	1436	MS, RI	Yes
**312**	*trans*-geranylacetone	3796-70-1	33.6	1442	1448-1456	MS, RI	Yes
**313**	Unknown101	NA	33.6	1442	NA	ND	
**314**	1-[2-(1-methylethylidene)cyclopropyl]ethanone	NA	33.7	1445	NA	MS	
**315**	Unknown102	NA	33.8	1447	NA	ND	
**316**	(*E*)-β-famesene	18794-84-8	33.8	1448	1457-1471	MS, RI	Yes
**317**	Unknown103	NA	34.0	1453	NA	ND	Yes
**318**	(5*Z*)-2,6,10-trimethyl-1,5,9-undecatriene	62951-96-6	34.1	1454	NA	MS	Yes
**319**	2,6,10-trimethyltridecane	3891-99-4	34.2	1457	1442-1465	MS, RI	
**320**	Unknown104	NA	34.3	1459	NA	ND	
**321**	Unknown105	NA	34.4	1461	NA	ND	
**322**	Pyroxyfur	70166-48-2	34.4	1463	NA	MS	
**323**	5,6-dihydro-6-pentyl-2H-pyran-2-one	54814-64-1	34.5	1465	1477-1520	MS, RI	
**324**	Unknown106	NA	34.6	1466	NA	ND	
**325**	Dehydro-β-ionone	1203-08-3	34.7	1469	1489	MS, RI	Yes
**326**	Unknown107	NA	34.8	1471	NA	ND	
**327**	*trans*-β -Ionone	79-77-6	34.8	1472	1469-1512	MS, RI	Yes
**328**	Unknown108	NA	34.9	1474	NA	ND	Yes
**329**	Eudesma-1,4(15),11-triene	212394-95-1	34.9	1474	1477	MS, RI	
**330**	Phenethyl 2-methylbutyrate	24817-51-4	35.0	1477	1484-1491	MS, RI	Yes
**331**	Methyl (*E*)-heptadec-10-en-8-ynoate	16714-85-5	35.1	1480	NA	MS	
**332**	Jasmine lactone	25524-95-2	35.2	1481	1517.8	MS, RI	Yes
**333**	Unknown109	NA	35.3	1484	NA	ND	
**334**	(*Z*,*E*)-α-farnesene	26560-14-5	35.4	1485	1462-1491	MS, RI	Yes
**335**	Unknown110	NA	35.5	1488	NA	ND	
**336**	1,2,4-trimethyl-3-nitrobicyclo[3.3.1]nonan-9-one	129967-65-3	35.5	1490	NA	MS	
**337**	Unknown111	NA	35.6	1491	NA	ND	
**338**	1-iodo-2-methylundecane	73105-67-6	35.7	1494	NA	MS	
**339**	Butylated hydroxytoluene	128-37-0	35.8	1495	1489-1517	MS, RI	
**340**	Unknown112	NA	35.9	1498	NA	ND	
**341**	α-farnesene	502-61-4	36.0	1500	1473-1522	MS, RI	Yes
**342**	2,4-di-tert-butylphenol	96-76-4	36.1	1502	1502-1512	MS, RI	
**343**	2-methyl-5-octyn-4-ol	60657-70-7	36.1	1504	NA	MS	Yes
**344**	*cis*,*cis*-5,9-tetradecadiene	NA	36.4	1510	NA	MS	

**345**	1-isopropyl-4,7-dimethyl-1,2,3,5,6,8α-hexahydronaphthalene	16729-01-4	36.4	1511	NA	MS	
**346**	n-tridecan-1-ol	112-70-9	36.4	1511	1556-1599	MS, RI	
**347**	5-hydroxy-3-methyl-1-indanone	57878-30-5	36.5	1513	NA	MS	
**348**	Dihydroactinidiolide	17092-92-1	36.7	1518	NA	MS	Yes
**349**	Oxalic acid, allyl dodecyl ester	959312-46-0	36.8	1521	NA	MS	
**350**	Unknown113	NA	36.9	1524	NA	ND	
**351**	Unknown114	NA	37.0	1526	NA	ND	
**352**	Unknown115	NA	37.1	1530	NA	ND	
**353**	Unknown116	NA	37.3	1534	NA	ND	
**354**	Hexanoic acid, phenylmethyl ester	6938-45-0	37.4	1537	1511-1549	MS, RI	
**355**	4-ethyl-tetradecane	55045-14-2	37.5	1541	1548	MS, RI	Yes
**356**	7-methylpentadecane	6165-40-8	37.7	1544	1539-1542	MS, RI	Yes
**357**	5-methylpentadecane	25117-33-3	37.8	1548	1546-1551	MS, RI	Yes
**358**	Unknown117	NA	37.9	1551	NA	ND	
**359**	Unknown118	NA	38.1	1555	NA	ND	
**360**	Nerolidol	7212-44-4	38.3	1561	1540-1575	MS, RI	Yes
**361**	5-iodo-nonane	59456-19-8	38.4	1563	NA	MS	
**362**	*cis*-3-hexenyl benzoate	25152-85-6	38.5	1565	1568	MS, RI	Yes
**363**	Unknown119	NA	38.5	1567	NA	ND	Yes
**364**	3-methylpentadecane	2882-96-4	38.6	1568	1569-1571	MS, RI	
**365**	(*3E*,*7E*)-4,8,12-trimethyltrideca-1,3,7,11-tetraene	62235-06-7	38.6	1569	NA	MS	
**366**	Unknown120	NA	38.7	1571	NA	ND	Yes
**367**	Dodecanoic acid, hex-3-enyl ester	NA	38.8	1573	NA	MS	
**368**	Benzoic acid, hexyl ester	6789-88-4	38.8	1573	1577-1599	MS, RI	Yes
**369**	Hematoporphyrin	14459-29-1	38.9	1577	NA	MS	
**370**	Hexyl octanoate	1117-55-1	38.9	1577	1580	MS, RI	Yes
**371**	Hexamethylene diacrylate	13048-33-4	39.0	1578	1599-1606	MS, RI	Yes
**372**	Unknown121	NA	39.0	1580	NA	ND	
**373**	(*E*)-2-hexenyl benzoate	76841-70-8	39.1	1580	1583	MS, RI	Yes
**374**	2,2,4-trimethyl-1,3-pentanediol diisobutyrate	6846-50-0	39.1	1582	1587	MS, RI	
**375**	Unknown122	NA	39.4	1590	NA	ND	
**376**	Unknown123	NA	39.5	1592	NA	ND	
**377**	Unknown124	NA	39.7	1598	NA	ND	
**378**	Unknown125	NA	39.7	1598	NA	ND	
**379**	Epicedrol	16230-29-8	39.8	1600	1593-1632	MS, RI	Yes
**380**	Unknown126	NA	40.0	1605	NA	ND	

**381**	*trans*-3,6-diethyl-3,6-dimethyl-tricyclo[3.1.0.0(2,4)]hexane	NA	40.3	1613	NA	MS	Yes
**382**	Unknown127	NA	40.4	1616	NA	ND	
**383**	Unknown128	NA	40.5	1616	NA	ND	
**384**	Unknown129	NA	40.6	1621	NA	ND	
**385**	Unknown130	NA	40.7	1623	NA	ND	
**386**	Unknown131	NA	40.7	1625	NA	ND	
**387**	Unknown132	NA	40.8	1627	NA	ND	
**388**	2-phenylethyl hexanoate	6290-37-5	41.1	1633	1650	MS, RI, Std	Yes
**389**	Unknown133	NA	41.1	1634	NA	ND	
**390**	Methyl (3-oxo-2-[(2Z)-2-pentenyl]cyclopentyl)acetate	42536-97-0	41.1	1634	NA	MS	Yes
**391**	T-muurolol	19912-62-0	41.2	1636	1620-1659	MS, RI	
**392**	Unknown134	NA	41.4	1641	NA	ND	
**393**	Unknown135	NA	41.5	1644	NA	ND	
**394**	(*E*)-α-cadinol	481-34-5	41.6	1647	1635-1669	MS, RI	
**395**	Unknown136	NA	41.7	1650	NA	ND	
**396**	Unknown137	NA	41.8	1653	NA	ND	
**397**	*cis*-3-hexenyl salicylate	65405-77-8	42.0	1658	1670	MS, RI	Yes
**398**	Unknown138	NA	42.1	1660	NA	ND	
**399**	2-bromononane	2216-35-5	42.2	1662	NA	MS	
**400**	1-(4-bromobutyl)-2-piperidinone	195194-80-0	42.3	1666	NA	MS	
**401**	Unknown139	NA	42.4	1668	NA	ND	
**402**	n-hexyl salicylate	6259-76-3	42.4	1668	1682-1683	MS, RI	
**403**	Unknown140	NA	42.6	1673	NA	ND	
**404**	Unknown141	NA	42.6	1674	NA	ND	
**405**	Unknown142	NA	42.7	1676	NA	ND	
**406**	1-hydroxycyclohexyl-1-phenyl methanone	947-19-3	42.7	1677	1687	MS, RI	
**407**	Unknown143	NA	43.0	1685	NA	ND	
**408**	Unknown144	NA	43.2	1690	NA	ND	
**409**	*cis*-3-hexenyl heptine carbonate	68698-58-8	43.3	1691	NA	MS	Yes
**410**	Unknown145	NA	43.4	1694	NA	ND	
**411**	Unknown146	NA	43.5	1698	NA	ND	
**412**	Unknown147	NA	43.7	1702	NA	ND	Yes
**413**	Farnesol	106-28-5	43.9	1707	1667-1749	MS, RI	
**414**	Unknown148	NA	43.9	1708	NA	ND	
**415**	2-methylhexanoic acid	4536-23-6	44.2	1718	NA	MS	
**416**	Unknown149	NA	44.2	1718	NA	ND	
**417**	Unknown150	NA	44.4	1722	NA	ND	
**418**	Unknown151	NA	44.6	1728	NA	ND	

**419**	Unknown152	NA	44.9	1738	NA	ND	Yes
**420**	Unknown153	NA	45.5	1756	NA	ND	
**421**	Phenanthrene	85-01-8	45.8	1763	1744-1784	MS, RI	
**422**	Oxalic acid, isobutyl pentyl ester	NA	45.9	1767	NA	MS	
**423**	Unknown154	NA	46.1	1771	NA	ND	
**424**	Unknown155	NA	46.6	1786	NA	ND	
**425**	Unknown156	NA	46.7	1789	NA	ND	
**426**	Unknown157	NA	46.9	1796	NA	ND	
**427**	Unknown158	NA	47.0	1799	NA	ND	
**428**	Caffeine	58-08-2	48.1	1832	1806-1848	MS, RI	Yes
**429**	Neophytadiene	504-96-1	48.2	1832	1836	MS, RI	Yes
**430**	6,10,14-trimethyl-2-pentadecanone	502-69-2	48.3	1836	1834-1864	MS, RI	Yes
**431**	Diisobutyl phthalate	84-69-5	48.7	1849	1845-1881	MS, RI	Yes
**432**	Unknown159	NA	49.0	1856	NA	ND	
**433**	Unknown160	NA	49.0	1857	NA	ND	
**434**	Unknown161	NA	49.4	1869	NA	ND	
**435**	3,5,11,15-tetramethyl-1-hexadecen-3-ol	649699-11-6	49.6	1874	NA	MS	Yes
**436**	Unknown162	NA	49.6	1876	NA	ND	
**437**	Unknown163	NA	50.2	1892	NA	ND	
**438**	Unknown164	NA	50.5	1901	NA	ND	
**439**	Unknown165	NA	50.7	1909	NA	ND	
**440**	Unknown166	NA	50.8	1911	NA	ND	
**441**	Hexadecanoic acid, methyl ester	112-39-0	51.1	1919	1894-1939	MS, RI	Yes
**442**	Isophytol	505-32-8	51.7	1940	1939	MS, RI	Yes
**443**	Phthalic acid, 4-bromophenyl octyl ester	NA	51.8	1943	NA	MS	
**444**	Unknown167	NA	53.2	1987	NA	ND	
**445**	Unknown168	NA	56.2	2083	NA	ND	
**446**	Methyl α-linolenate	301-00-8	56.3	2088	2096	MS, RI	
**447**	Phytol	150-86-7	56.7	2100	2107	MS, RI	Yes

Note: MS, mass spectrum comparison using NIST libraries; RI, the retention index compared with the literature value; Std, mass spectrum compared with the chemical reference standard; NA, not available; ND, not determined.

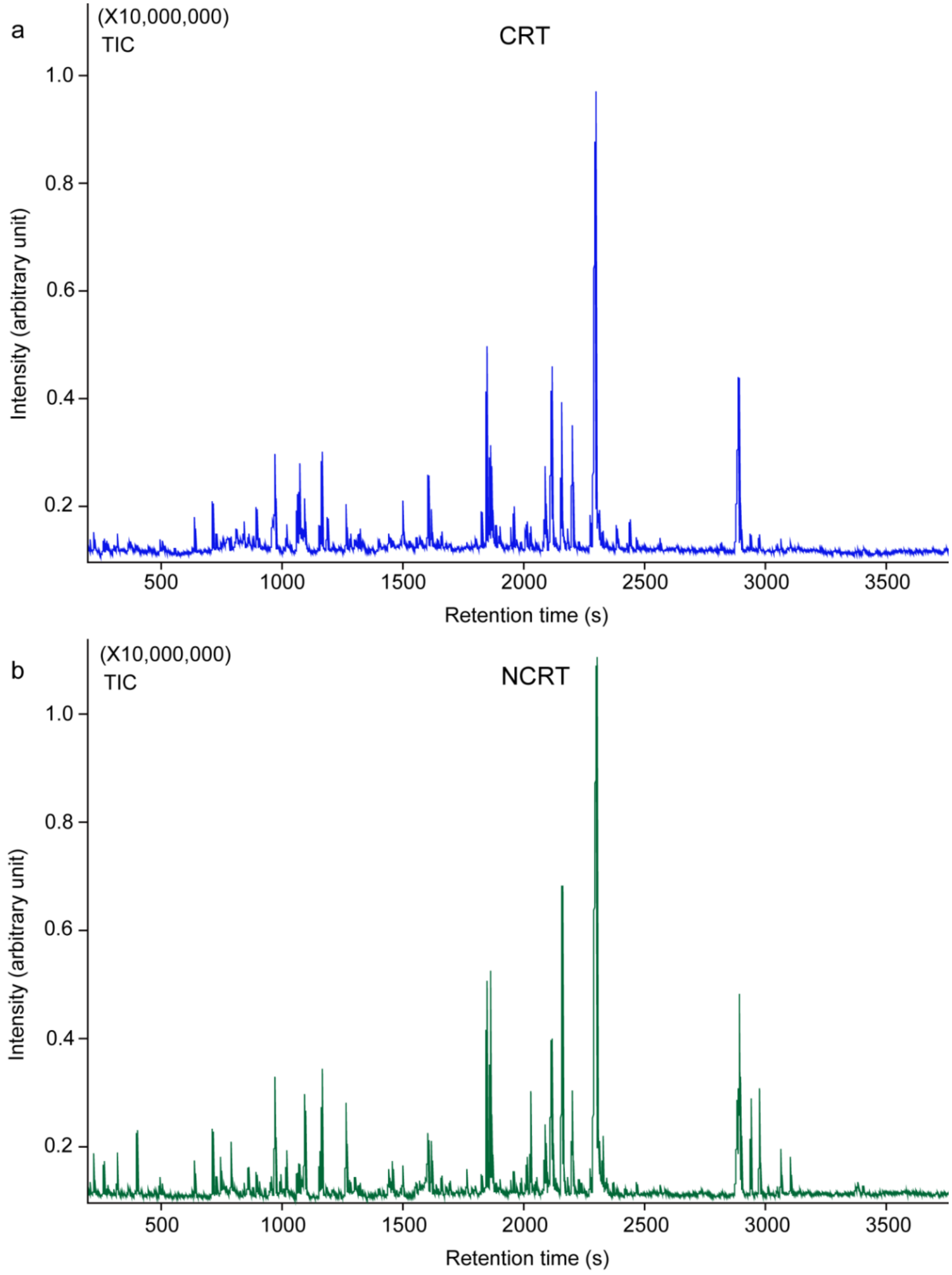

Figure S1. Representative total ion chromatograms (TICs) of CRT (A) and NCRT (B) Wuyi rock tea samples.

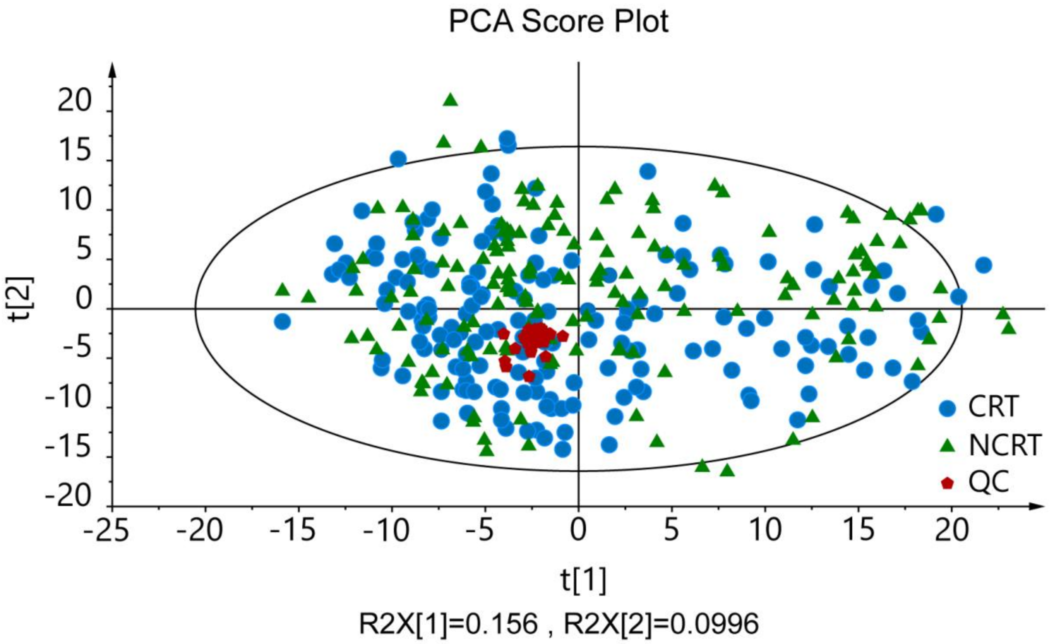

Figure S2. PCA score plot showing clustering of pooled QC samples.
